# Direct Enantiomer Differentiation of Drugs and Drug-Like Compounds via Noncovalent Copper–Amino Acid Complexation and Ion Mobility-Mass Spectrometry

**DOI:** 10.1021/acs.analchem.4c02710

**Published:** 2024-07-25

**Authors:** Benjamin K. Blakley, Emanuel Zlibut, Rashi M. Gupta, Jody C. May, John A. McLean

**Affiliations:** Department of Chemistry, Center for Innovative Technology, Vanderbilt Institute of Chemical Biology, Vanderbilt-Ingram Cancer Center, and Vanderbilt Institute for Integrated Biosystems Research and Education, Vanderbilt University, Nashville, Tennessee 37235-1822, United States

## Abstract

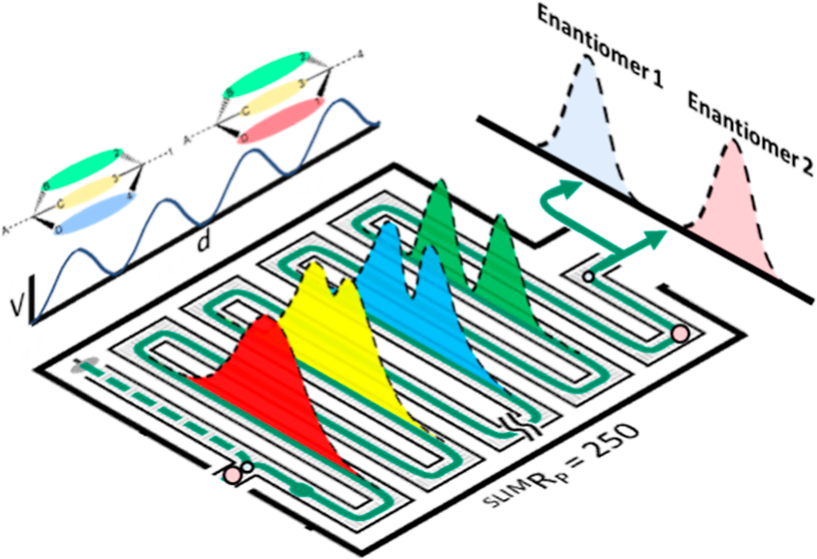

Drug enantiomers can possess vastly different pharmacological properties, yet they are identical in their chemical composition and structural connectivity. Thus, resolving enantiomers poses a great challenge in the field of separation science. Enantiomer separations necessitate interaction of the analyte with a chiral environment—in mass spectrometry-based analysis, a common approach is through a three-point interaction with a chiral selector commonly introduced during sample preparation. In select cases, the structural difference imparted through noncovalent complexation results in enantiomer-specific structural differences, facilitating measurement using a structurally selective analytical technique such as ion mobility-mass spectrometry (IM-MS). In this work, we investigate the direct IM-MS differentiation of chiral drug compounds using mononuclear copper complexes incorporating an amino acid chiral selector. A panel of 20 chiral drugs and drug-like compounds were investigated for separation, and four l-amino acids (l-histidine, l-tryptophan, l-proline, and l-tyrosine) were evaluated as chiral selectors (CS) to provide the chiral environment necessary for differentiation. Enantiomer differentiation was achieved for four chiral molecule pairs (*R*/*S*-thalidomide, *R*/*S*-baclofen, *R*/*S*-metoprolol, and d/l-panthenol) with two-peak resolution (*R*_p–p_) values ranging from 0.7 (>10% valley) to 1.5 (baseline separation). Calibration curves relating IM peak areas to enantiomeric concentrations enabled enantiomeric excess quantitation of racemic thalidomide and metoprolol with residuals of 5.7 and 2.5%, respectively. Theoretical models suggest that Cu^II^ and l-histidine complexation around the analyte chiral center is important for gas-phase stereoselectivity. This study demonstrates the potential of combining enantioselective noncovalent copper complexation with structurally selective IM-MS for differentiating chiral drugs and drug-like compounds.

## Introduction

There is considerable interest in developing high-throughput methods to differentiate drug enantiomers.^1^ From a pharmaceutical perspective, drug enantiomers can have drastically different effects on the human body: typically, one chiral isomer exhibits a desired therapeutic effect (the eutomer), whereas its enantiomer (the distomer) can produce no effect, diminished effects, or even severe toxic effects on the human body.^1,2^ Additionally, designing enantiospecific synthetic routes to a drug is challenging, and many drug synthetic routes used today result in a racemic mixture of the two chiral forms.

From an analytical perspective, enantiomers exhibit few physicochemical differences that allow separation and differentiation, and thus chiral compounds are among the most challenging class of stereoisomers to resolve.^3,4^ Predominant analytical methods for enantiomer differentiation include chiral liquid chromatography (LC),^5−7^ nuclear magnetic resonance (NMR) spectroscopy,^8−10^ capillary electrophoresis (CE),^11−13^ and tandem mass spectrometry (MS/MS).^14−16^ In each of these techniques, a chiral environment is necessary to achieve differentiation.

Another analytical method for differentiating enantiomers is tandem MS/MS. Notably, MS/MS analysis of chiral analytes was achieved using the kinetic method, pioneered by Cooks and co-workers in the late 1990s. In the kinetic method, chiral ligands form ternary complexes with a CS and transition metal in the +2 oxidation state. This results in two diastereomeric complexes that dissociate at different MS/MS energies which can be used for chiral differentiation and quantitation of enantiomeric excess (ee).^14−16^ As an MS-based method, the kinetic method can perform with high sensitivity but is generally limited by low throughput with analysis times on the order of hours, as dictated by the energy-resolved MS/MS acquisition, as well as operating with limited structural resolution associated with the energy resolution of the tandem MS/MS stage. Additionally, not all chiral compounds form diastereomeric complexes with the metal and CS; thus, the kinetic method is selective to only certain drug analytes.^5,17−19^

A noteworthy recent method, published in 2024 by Ouyang and co-workers, utilized an ion trap MS to differentiate enantiomers using “symmetry breaking motions” in a rotating electric field.^20^ Notably, this method achieved direct differentiation of gas-phase small molecule enantiomers without the use of chiral modifiers. However, the precise relationship between chirality and directional rotation is not presently fully understood—and this approach has yet to be demonstrated on other MS instrumentation.

A high-throughput and sensitive method for drug enantiomer differentiation with a broad applicability to different classes of drugs is of high interest to analytical chemists. Ion mobility spectrometry (IM) is a structurally selective analytical technique that offers millisecond-scale gas-phase separation based on ion size and shape, which are captured in the measured collision cross section (CCS) value.^21,22^ When IM is coupled to mass spectrometry (IM-MS), differentiation of isobaric and isomeric analytes can be achieved on the basis of molecular size and weight.^3,23,24^ For instance, a body of work investigating peptide epimer separations using IM-MS has been published by Li and co-workers.^25−27^ These works collectively demonstrate the utility of IM-MS for the structural characterization and site-specific localization of d-amino acids in diastereomeric peptide chains. However, small molecule enantiomers are identical in atomic composition and connectivity and thus do not exhibit CCS differences that can lead to resolution at any IM resolving power.^3^

A strategy that has demonstrated success with IM is to measure the imparted structural differences of diastereomeric complexes formed between the chiral analyte and a chiral shift reagent.^4,7^ Common chiral shift reagents include cyclodextrins,^13,28^ derivatized crown ethers,^29,30^ self-associated multimers,^31,32^ transition metal-amino acid (AA) complexes,^33−36^ and steroids.^37^ Inclusion-based shift reagents (i.e., cyclodextrins and crown ethers) have demonstrated reproducible IM separations, but the host cavity must be of sufficient size to exhibit selectivity via inclusion.^29,38^ Thus, for broad enantiomeric selectivity, bidentate ligands such as transition metal-amino acid shift reagents that take advantage of three point interactions should be less sensitive to analyte size. It is known that chiral recognition is affected by transition metal properties, including d-orbital occupancy and splitting, as well as the hard–soft acid–base theory.^39^ Divalent copper (Cu^II^) is frequently used due to its high affinity for aromatic amino acids such as histidine, which spontaneously form a Cu–His complex,^40^ as well as an observed higher degree of separation compared to other divalent metals such as nickel, zinc, calcium, and magnesium.^41^

The first direct observation of enantioselective transition metal-bound complexes using IM-MS was demonstrated for amino acid enantiomers in 2007.^41^ Since then, several papers have been published demonstrating amino acid enantiomer differentiation using copper complexes.^34,35,42^ However, less work has been reported for drug enantiomer differentiation using noncovalent complexation and IM-MS. Mie et al. published the separation and quantification of terbutaline enantiomers in 2008 using a mononuclear copper [(M)(AA)_2_(Cu^II^)–H]^+^ complex and FAIMS-MS/MS.^17^ In other studies, cyclodextrin bound to a metal cation has been used to differentiate enantiomers of penicillamine^43^ and ibuprofen.^38^ In general, drug enantiomer separation has been limited by several factors, including the relatively low resolving powers (<100) associated with earlier generation IM technologies. In recent years, several high resolution ion mobility (HRIM) instruments capable of resolving powers in excess of 200 have been released, including trapped IM (TIMS),^44^ cyclic multipass TWIMS (cIM),^48^ and extended path-length structures for lossless ion manipulations operated with traveling waves (TWSLIM).^45−47,49^ In particular, TWSLIM-MS is an emerging technology which utilizes traveling wave IM separations over a long (13 m) serpentine path length in order to achieve IM resolving powers of 200 or more across a wide mass and mobility range.^45−47,50^ In 2018, Nagy et al. utilized an ultralong (58.5 m) SLIM to separate amino acid enantiomers using cyclodextrin inclusion.^28^ The pairing of chirally selective shift reagents with enhanced structural resolution afforded by newly emerging HRIM technologies represents a powerful development in the field of drug enantiomer separations.

**Figure 1:**
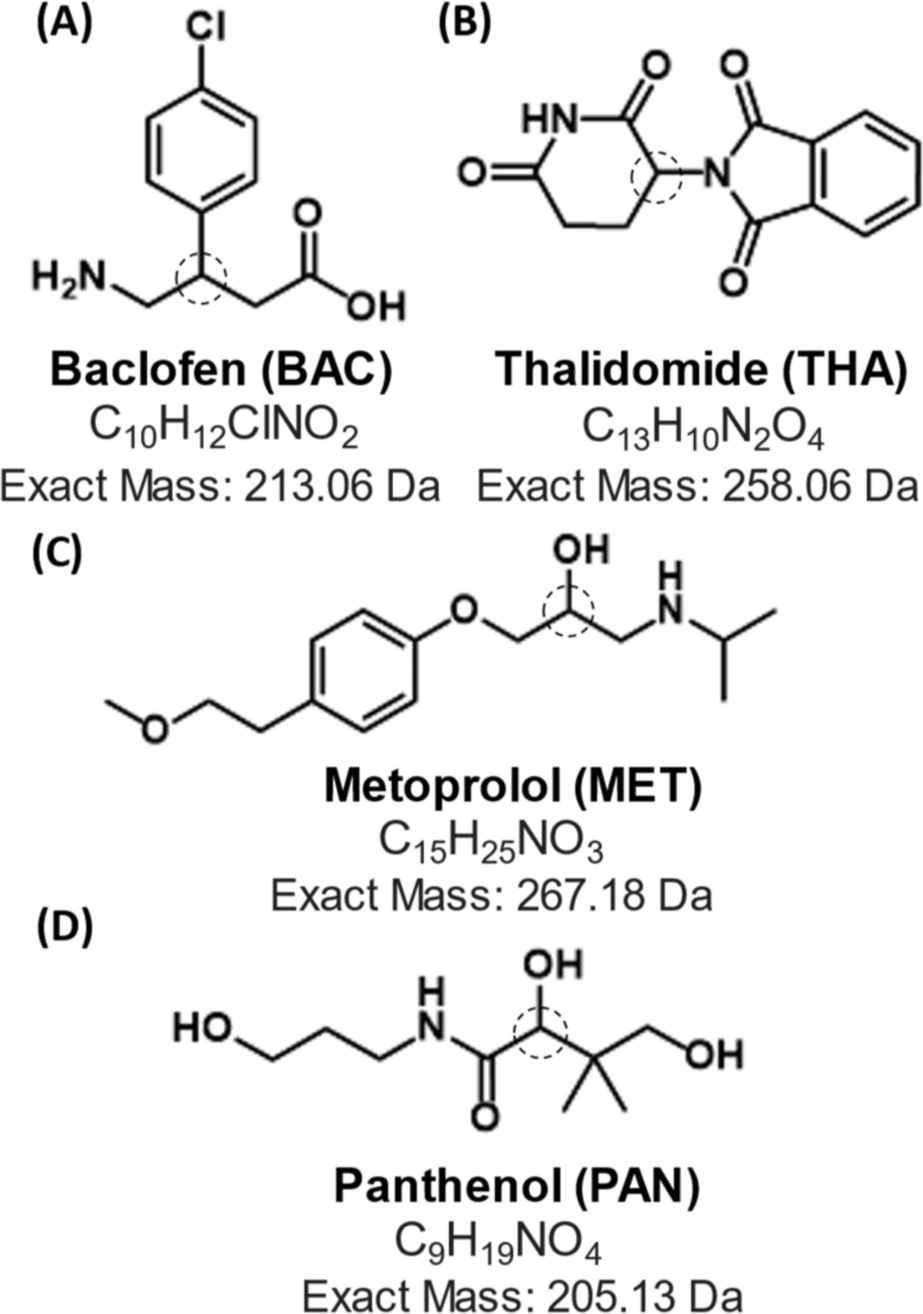
Structures, molecular formulas, and exact monoisotopic masses for chiral compounds that were successfully separated by chirally selective IM-MS, including (A) baclofen, (B) thalidomide, (C) metoprolol, and (D) panthenol. Chiral centers are denoted with dashed circles.

Here, we investigate a CS approach using copper–amino acid-bound analyte complexes toward screening a large (*n* = 20) panel of drugs and drug-like chiral molecules using both conventional resolution IM-MS and HRIM-MS. To determine the general applicability of this approach, priority is placed on ternary complexes of the form [(M)(AA)(Cu^II^)–H]^+^ as a means to differentiate small-molecule drugs and drug-like enantiomers. Results demonstrate that these mononuclear complexes are highly effective chirally selective modifiers which impart CCS differences that are measurable with conventional resolution IM-MS and directly resolvable by HRIM-MS. This enabled direct differentiation of four racemic drugs and drug-like compounds (Figure 1) and allowed for the quantitation of ee, as demonstrated for thalidomide and metoprolol mixed enantiomer samples. Theoretical DFT and molecular dynamics-based computational results provided structural insights into observed separations to enable the interpretation of the experimental findings.

## Experimental Methods

### Standards and Chemicals

Optically pure amino acids, drugs, and drug-like compounds, as well as racemic mixtures, were obtained from various vendors. These compounds included baclofen, thalidomide, metoprolol, and panthenol. High-purity (Optima LC–MS grade) methanol and water were obtained from Fisher Scientific, and copper(II) acetate was purchased from Sigma-Aldrich. All chemicals were used as received. A full summary of reagent and vendor sources can be found in Table S1. Corresponding structures for each reagent can be found in Figure S1.

### Sample Preparation

A 1 mg/mL stock solution of each compound was first prepared in 50:50 methanol/water. Samples for IM-MS analysis were created by combining equimolar aliquots of copper acetate and a chiral selector stock solution with a given analyte stock. Samples were then diluted using 50:50 methanol/water to final concentrations of 20 μM (DTIMS) and 100 μM (TWSLIM). The increase in sample concentrations for TWSLIM analysis was used to offset a decrease in sensitivity, which we presume is due to higher energy ion transfer conditions in our TWSLIM method which results in a higher degree of dissociation for noncovalent complexes.

### Instrument Parameters

Samples were directly infused (10 μL/min) into an ESI source (Jet Stream, Agilent Technologies, Santa Clara, CA) coupled to one of two IM-MS instruments (Figure S2): a drift tube IM-MS (DTIMS, 6560 IM-QTOF, Agilent) or a SLIM-based traveling wave HRIM-MS (TWSLIM-MS, MOBIE, MOBILion Systems, Chadds Ford, PA) interfaced with a quadrupole-time-of-flight mass spectrometer (6546, Agilent). Both instrument platforms were operated in positive ionization mode with the QTOF tuned to a low-mass range. ESI was operated with a gas flow rate of 8 L/min at a temperature of 325 °C. The capillary voltage was operated at 2.2 kV, and the nozzle voltage was operated at 2.0 kV. DTIMS method parameters were set to conditions previously published by Zlibut et al.^34^ TWSLIM parameters were optimized for the transmission of mononuclear copper complexes, which notably involved decreases in fragmentor voltage, separation TW amplitude, and separation TW frequency. A full description of TWSLIM-MS method parameters used to optimize transmission and survival of noncovalent complexes can be found in Table S2, and a comparison of MS results from the default and optimized methods can be found in Figure S3. Ternary complexes with various stoichiometries were identified based on mass accuracy (±10 ppm) and isotope distributions that reflected the characteristic isotopic envelope of copper (Figure S4).

### Data Processing and Software

For TWSLIM-MS data, PNNL PreProcessor (3.0) was utilized for three-point moving average smoothing and ^TWSLIM^CCS_N2_ drift axis conversion.^51^ For both DTIMS and TWSLIM, ion mobility data were manually extracted in Agilent MassHunter IM-MS Browser (version 10.0.1.10039). ^DT^CCS_N2_ conversion, RSD, and two-peak resolution (*R*_p–p_) calculations were performed in Microsoft Excel. TWSLIM peak areas were determined via simple integration using Agilent MassHunter Qualitative Analysis (version 10.0.10305.0), which does not perform any fitting or deconvolution.

### Experimental CCS Calculations

DTIMS-based arrival time measurements were converted to ^DT^CCS_N2_ values by using the single-field CCS calibration method. This method uses symmetrically branched hexakis(fluoroalkoxy)-phosphazene calibrant ions (HFAPs, Agilent Tune Mix) to generate a linear equation relating arrival time to CCS which is based on the first-principles fundamental ion mobility equation, commonly referred to as the Mason–Schamp relationship.^52,53^Equation 1 depicts the single-field calibration equation
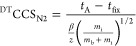
1Here, *t*_A_ is the DTIMS-measured arrival time, *m*_i_ is the mass of the analyte ion, *m*_b_ is the mass of the background drift gas (in this case, nitrogen), and β and *t*_fix_ are experiment-specific coefficients generated from calibration.

Because of the complicated relationship between TW parameters and the measured arrival time, a simple linear calibration equation cannot be used.^54^ Previous TWSLIM work found that a third-order polynomial calibration equation yielded the lowest CCS error when compared with both power fit and second-order polynomial.^50,55,56^ Thus, for ^TWSLIM^CCS calculations, a third-order polynomial was used to convert the TWSLIM-based arrival time measurements to CCS values. Equation 2 shows the functional form of the third-order polynomial utilized for TWSLIM-based CCS calibration^56^
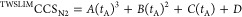
2where *A*, *B*, *C*, and *D* are experiment-specific coefficients generated from calibrants measured under the same conditions as the analytes to be calibrated.

### Assessment of Enantiomer Differentiation

Drug enantiomer differentiation was quantified using peak-to-peak resolution (*R*_p–p_), calculated in CCS space using eq 3(57)
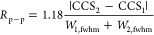
3

CCS_1_ and CCS_2_ refer to the measured collision cross section of each drug enantiomer (the *R* and *S* forms), and *W*_1_ and *W*_2_ are the corresponding peak full width measured at half its maximum height (fwhm). For reference, two peaks are considered unresolved (0%) at an *R*_p–p_ < 0.50, 10% resolved at 0.61, 50% at 0.83, 90% at 1.23, and baseline (100%) separated at >1.50.^57^

### Calculation of Enantiomeric Excess

Sample-specific enantiomer composition was quantified using ee, a value reflecting how much more of a given enantiomer is present in a sample than in its chiral counterpart. The ee was calculated from the concentration of each enantiomer present in a given sample by using eq 4
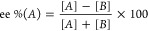
4

In a given sample, [*A*] corresponds to the molar concentration of the enantiomer whose complex arrives first in TWSLIM (that is, the peak with the smaller CCS), and [*B*] corresponds to the enantiomer with the larger CCS. To illustrate, a purely racemic mixture (50/50) of thalidomide would exhibit an ee(*S*) of 0%. Additionally, the *S*-enantiomer of thalidomide, when chelated within a mononuclear copper complex, has a smaller CCS than its *R* counterpart. Thus, a thalidomide sample containing 80 μM *S*-enantiomer and 20 μM *R*-enantiomer should exhibit an ee %(*S*) value of 60%.

### Computational Modeling and Theoretical CCS Calculations

Computational modeling was used to aid in interpreting the stereoselective gas-phase conformations contributing to IM separation. Structures for the mononuclear complex ions of the form [(M)(l-His)(Cu^II^)–H]^+^ were modeled as previously described.^58^ Projection approximation (PA) mobility calculations for all theoretical structures were performed in helium using MOBCAL software. For our computational workflow, we generate 3000 theoretical structures for each complex. Because the CCS is calculated for each theoretical structure, PA was selected for its quick and relatively accurate CCS calculation, as determined from the previous work.^58^ This procedure was performed for mononuclear copper complexes containing *R*/*S*-thalidomide, *R*/*S*-metoprolol, *R*/*S*-baclofen, and *R*/*S*-panthenol. The 3000 theoretical structures were plotted plotted, and the 600 lowest-energy structures were clustered by similarity using a root-mean-square distance analysis, and representative average structures are generated for each simulated structure. Drift tube CCS measurements obtained in helium drift gas (^DT^CCS_He_) have been previously shown to exhibit good correlation to computational results due to the minimal contribution of helium to the experimental CCS. Thus, to align computational results, ^DT^CCS_He_ measurements were taken for each system under computational study.

## Results and Discussion

### Enantiomer Differentiation via HRIM

**Figure 2:**
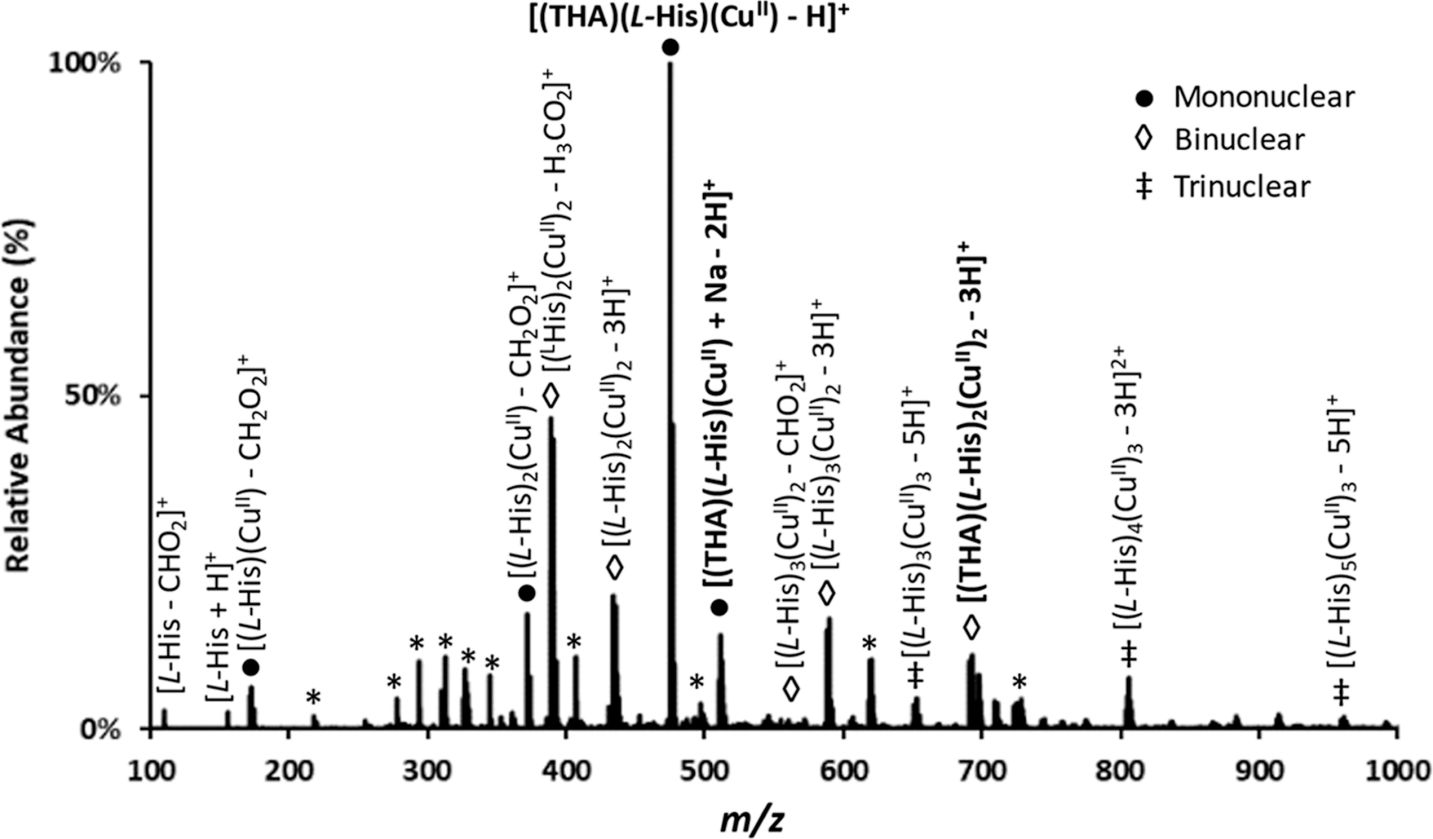
Example mass spectrum of a sample containing racemic thalidomide (M = thalidomide), l-histidine, and copper(II) acetate. Peak annotations are shown for ions containing copper at expected stoichiometries, with the number of copper atoms indicated by symbols. Annotations in bold indicate ternary complexes incorporating copper, histidine, and thalidomide. Asterisks (*) denote unidentified peaks with isotope distributions that do not indicate the presence of copper or exhibiting mass defects that do not correspond to peaks of interest.

**Figure 3:**
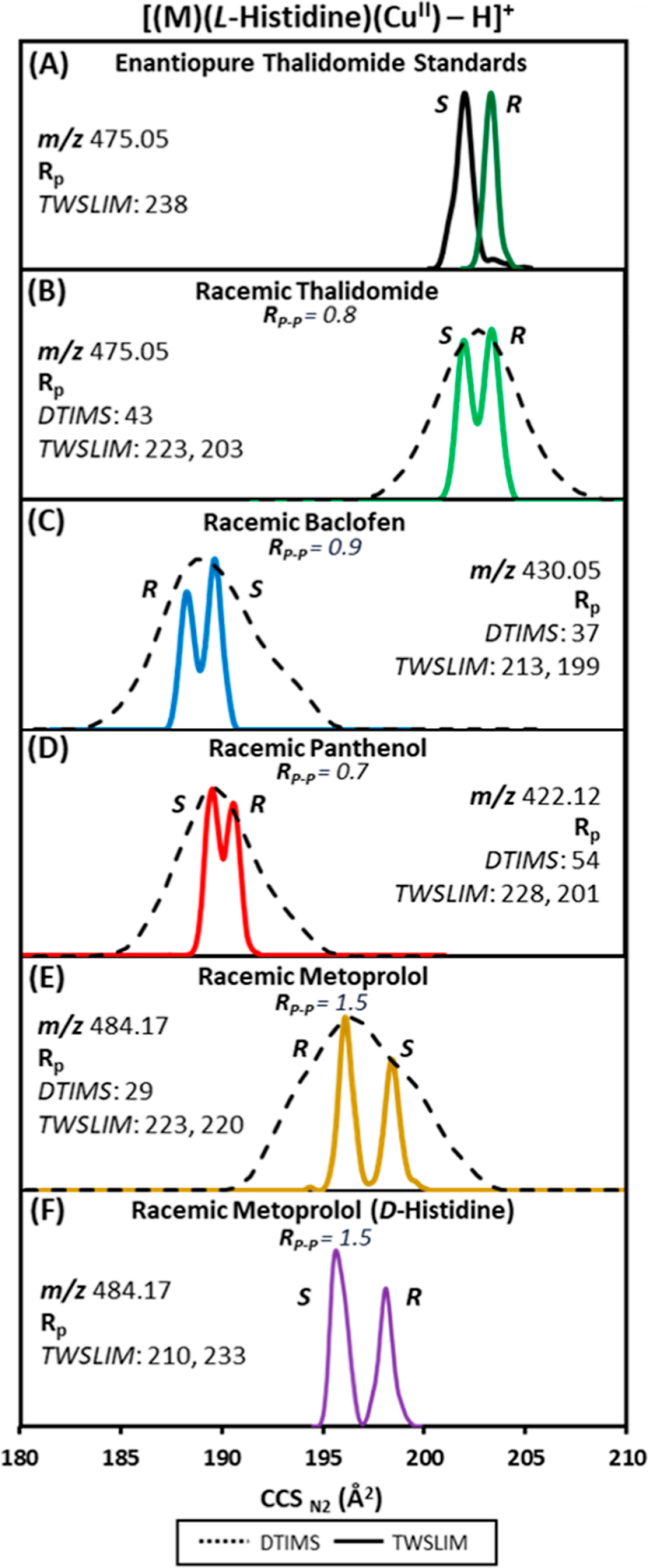
TWSLIM-MS spectra for (A) *S*- and *R*-thalidomide, (B) racemic thalidomide, (C) racemic baclofen, (D) racemic panthenol, and (E) racemic metoprolol. Single-peak resolving power (*R*_p_) and two-peak resolution (*R*_p–p_) are shown for each profile. The first TWSLIM *R*_p_ describes the first peak (i.e., the smaller CCS). TWSLIM data is shown as solid lines and DTIMS data is shown as dashed lines, respectively. For consistency, d- and l-panthenol are labeled *R* and *S*, respectively. (F) Results for metoprolol using d-histidine.

As summarized in Figure 2, initial MS studies observed the mononuclear [(M)(AA)(Cu^II^)–H]^+^ complex at especially high abundance, prompting interest in this simpler stoichiometry over higher order forms (e.g., bi- and trinuclear). While we have not rigorously investigated the effects of varying ratios of different components, we note that we have observed generally that altering the relative concentrations of copper, amino acid, and drug did not have a significant effect on the formation of ternary copper complexes under investigation. An initial DTIMS screening of the 20 racemates resulted in prioritization of 13 compounds for further HRIM analysis via TWSLIM. See the Supporting Information for more information about chiral selector and target complex optimization (Table S3 and Figure S5) and racemic compound screening (Table S4 and Table S5). Because dexamethasone and betamethasone are diastereomers (and not enantiomers), their separation was not analyzed via TWSLIM, although we note that the mononuclear complex was observed in the screening. Of the 12 corresponding mononuclear complexes analyzed via TWSLIM-MS, four were found to exhibit differentiation (where the racemic mixture exhibited an *R*_p–p_ > 0.5): thalidomide, baclofen, panthenol, and metoprolol (Figure 3B–E). Varying the chirality of histidine results in inversion of the TWSLIM arrival order but does not affect the TWSLIM profile. This is shown in Figure 3F, where incorporation of d-histidine results in *S*-metoprolol having a smaller CCS than *R*-metoprolol—the reverse is observed for l-histidine, as noted for amino acids in 2022 by Zlibut et al.^34^ However, for both panels, the more compact gas-phase structure is observed at higher intensity than its higher CCS counterpart, and the approximate ratios of the two peaks remain constant. This conservation of TWSLIM profile features was observed for each drug showing separation using l-histidine. d-histidine TWSLIM profiles can be found in Figure S6. Interestingly, of the investigated complexes incorporating l-histidine, half exhibited a smaller CCS when the *R*-analyte was complexed, while the other half exhibited a more compact structure for the *S*-analyte complex.

In order to visualize the effects of higher resolving power on racemate enantiomer differentiation, both DTIMS and TWSLIM spectra were overlaid in CCS space in Figure 3 panels (dashed vs solid lines, respectively). TWSLIM resolving powers were consistently observed around 220—because TWSLIM operates with higher resolving power, TWSLIM peaks are narrower than their DTIMS counterparts. A high degree of differentiation was achieved for racemic metoprolol with an *R*_p–p_ of 1.5 (baseline separation). Additionally, >50% differentiation was observed for both baclofen (*R*_p–p_ = 0.9) and thalidomide (*R*_p–p_ = 0.8). While panthenol was resolved with only an ∼10% valley (*R*_p–p_ = 0.7), this modest separation is still sufficient for quantitative determination of ee.^34^Table S6 contains interday replicate data for mononuclear [(M)(l-His)(Cu^II^)–H]^+^ complexes of thalidomide, baclofen, panthenol, and metoprolol, and Table S7 shows ^TWSLIM^CCS_N2_ measurements for d-histidine complexes. Table S8contains a comprehensive list of ^TWSLIM^CCS_N2_ and *R*_p–p_ values measured for each target complex exhibiting differentiation. To align the DTIMS and TWSLIM spectra, a single correction factor (∼1 Å^2^) was applied to all ^TWSLIM^CCS_N2_ values obtained from calibration, which is consistent with findings from Rose et al.^56^ Resolving power and two-peak resolution values calculated in-house were validated using peak fitting in AIST Software’s PeakLab (Figure S7)—these results were consistent with in-house calculations.

Enantiopure results for thalidomide (Figure 3A) aligned well with each of the two peaks observed from the racemic results (Figure 3B). This indicates that the two TWSLIM peaks correspond to *S*- and *R*-thalidomide and confirms the IM arrival order of *S* first and then *R* second. Enantiopure standards of metoprolol also confirmed the identity of the TWSLIM peaks obtained from the corresponding racemic samples. Only one pure enantiomer of panthenol and baclofen was commercially available (d-panthenol and *R*-baclofen, respectively), but this was sufficient for assigning *R* and *S* designations for their corresponding racemic TWSLIM spectra. DTIMS and TWSLIM spectra for three mononuclear racemate complexes where IM separation was not observed (i.e., flurbiprofen and chloramphenicol) can be found in Figure S8—these two examples are representative of the other compounds where differentiation was not observed.

### Determination of Enantiomeric Excess via HRIM

**Figure 4:**
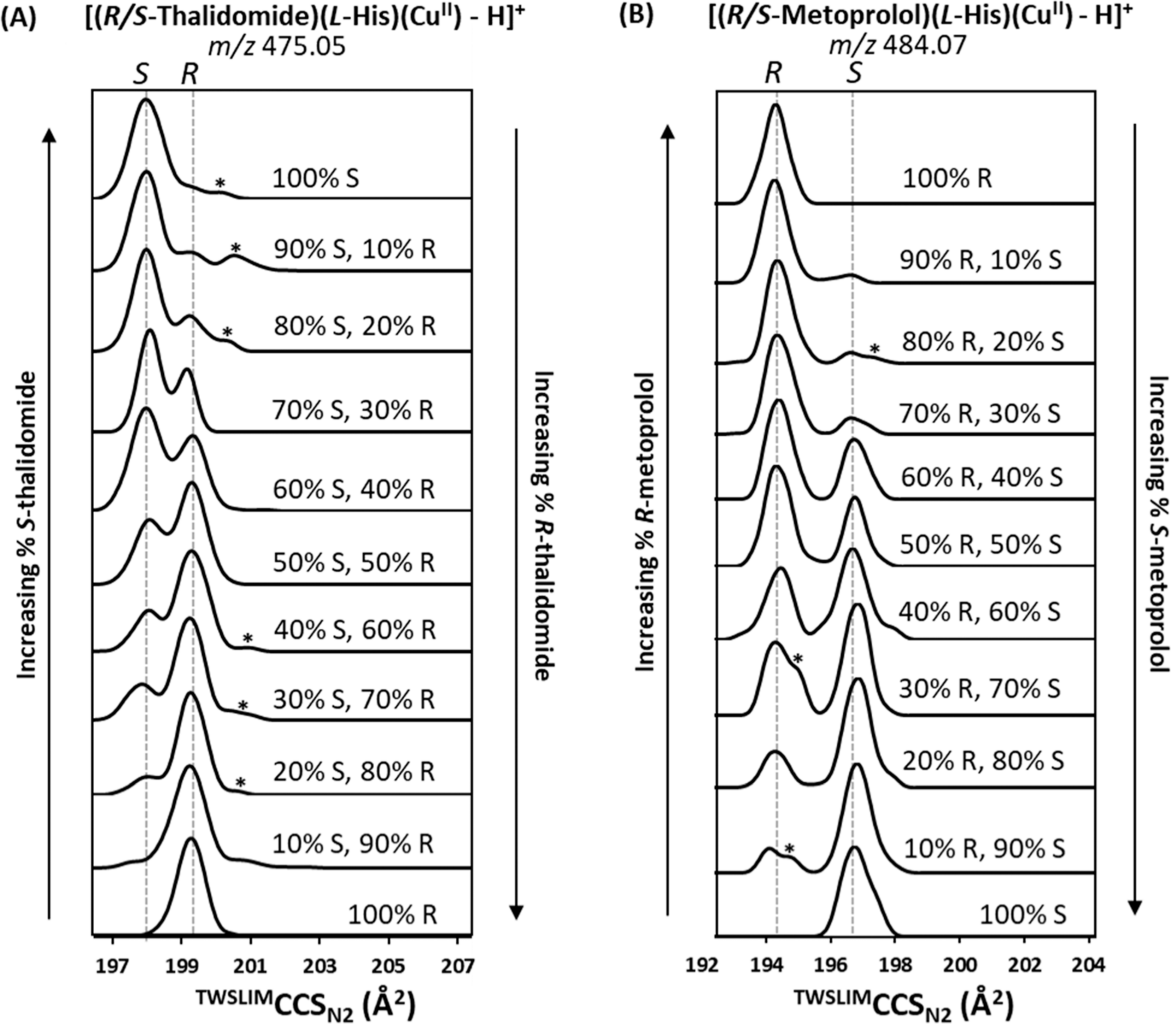
[(M)(l-His)(Cu^II^)–H]^+^ TWSLIM profiles at varying ee for (A) *R*/*S*-thalidomide and (B) *R*/*S*-metoprolol. Features arising from tailing in (A) and from low intensity in (B) are marked with an asterisk (*).

Pure enantiomer standards were available for thalidomide and metoprolol, allowing for an evaluation of the ee. Sample mixtures of the pure enantiomers were prepared with different molar ratios of known ee. Each sample mixture was then mixed with copper(II) acetate and l-histidine and subsequently analyzed via TWSLIM-MS. The resulting mononuclear HRIM profiles are summarized in Figure 4 and demonstrate an expected shift in the relative abundance of the two IM profiles in response to changes in the molar ratios of each enantiomer. Each peak was integrated, and the peak area ratio (*r*) was obtained via eq 5
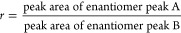
5

The peak area of enantiomer peak A refers to the TWSLIM peak with the smaller ^TWSLIM^CCS_N2_ value, while peak B refers to the larger ^TWSLIM^CCS_N2_ value. For thalidomide, peak A is the *S* enantiomer, and for metoprolol, peak A is the *R* enantiomer, as determined from analyzing the corresponding enantiopure standards. We note here that the additional features observed for several profiles in Figure 4B (e.g., 30% *R*, 10% *R*, and 20% *S*) is thought to be the result of low intensity associated with the minor peak in each of these distributions. Similarly, the additional feature observed at the end of many spectra in Figure 4A is thought to be the result of tailing. These *r* values were plotted against 1/(100 – ee_sample_) and fitted to a linear equation to generate a calibration relationship (Figure S9). A list of the resulting ee measurements can be found in Tables S9–S11. The resulting calibration relationship exhibited excellent linearity (*R*^2^ = 0.9994 for thalidomide and 0.9863 for metoprolol) and minimal interday variability. This is especially notable due to the small ^TWSLIM^CCS_N2_ differences exhibited by the two copper complexes (approximately 0.67% for thalidomide and 1.18% for metoprolol). The high error exhibited by the thalidomide 80 ee sample and metoprolol 60 and 80 ee samples is thought to be due to sensitivity issues at low concentrations as was also observed in similar studies.^35^ The optimized ion settings used to transport these intact complexes operate with reduced transmission efficiency, which likely contributes to this error.

To evaluate the validity of the calibration relationship, two tests were performed. First, the ratio corresponding to each measurement was applied to the calibration curve equation to obtain the predicted ee for each point. The residual was then calculated via eq 6
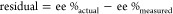
6

**Figure 5:**
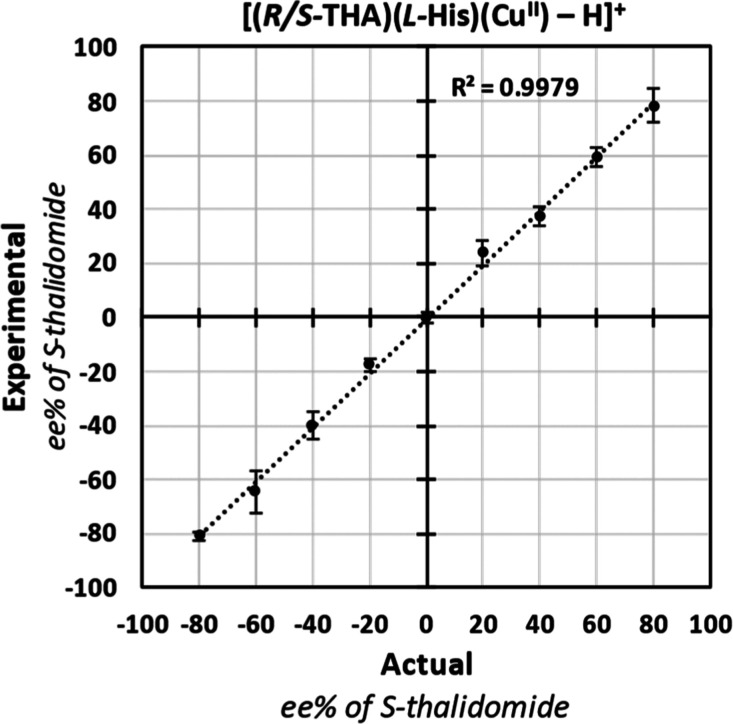
Plot of the experimental ee vs the actual ee for thalidomide samples. Points on the graph represent the average of three interday measurements. Error bars depict standard deviations of interday measurements.

Figure 5 is a plot comparing thalidomide’s measured ee to the actual ee. Each point is the average of three interday replicates, and error bars correspond to the interday standard deviation. The measured ee showed close correlation to actual ee values (*R*^2^ = 0.9979). Additionally, low standard deviations were observed for each measurement. Metoprolol exhibited consistently higher residuals at low *R*/*S* ratios (e.g., 1 to 9, 2 to 8, and 3 to 7); however, as the ratio increased, residuals decreased to values comparable with thalidomide data. As a second test, the calibration relationship was applied to racemic samples of both drugs. Racemic samples are expected to have an ee of 0 by definition. An error of 5.7% was obtained for racemic thalidomide, which was consistent with published results quantifying 1:1 mixtures of drug enantiomers using the kinetic method.^15^ The same calculation was performed for racemic metoprolol, which resulted in a measured ee of 2.46%.

There is literature precedent for the racemization^59^ and chiral inversion^60^ of thalidomide enantiomers, where it was found that chiral inversion and degradation leads to an average steady-state thalidomide *R*/*S* concentration ratio of 1.07 after 12 h incubation in blood.^60^ This suggests that, in addition to interconversion, other factors such as sample impurities are further indicated by data in Figure 4, where the 50:50% *S*/*R* thalidomide TWSLIM spectrum exhibits a higher *R* peak than *S* peak. The *R*/*S* peak area ratio of this IM profile was determined to be 1.61—a value considerably higher than 1.07. A similar phenomenon was observed by Cooper-Shepherd et al. in their investigation of thalidomide enantiomer differentiation using self-association dimers.^31^ In this study, a cIM peak attributed to heterochiral (*R*,*S*)/(*S*,*R*) thalidomide dimers was also observed in the enantiopure *S*-thalidomide sample. The authors of the paper attribute this observation to impurities in their *S*-thalidomide sample, and we note here that the impurity they observe could be further attributed to chiral inversion.

### Structural Insights from Theory

Conformational space plots projecting CCS_He_ and relative energy for 3000 theoretical structures corresponding to the mononuclear complexes of the form [(M)(l-His)(Cu^II^)–H]^+^ for thalidomide, baclofen, metoprolol, and panthenol are shown in Figure S10. A full summary of experimental helium CCS measurements can be found in Table S12. Experimental CCS measurements in helium align to the low-energy portion of these plots, as previously noted.^34,58,61^ Alongside each conformational space plot are representative average structures of the 600 lowest-energy conformations. The atomistic models obtained from theory suggest that stereoselectivity is achieved based on the specific orientation of Cu^II^ and l-His around the chiral center. In each case, the relative orientation of each chiral center leads to one enantiomer adopting a more compact conformation, sufficient to be resolved by HRIM.

Modeling results show that metoprolol enantiomers (Figure S10C) exhibit the most structurally different orientation around the chiral center, while panthenol (Figure S10D) exhibits more structurally similar orientations. This is consistent with TWSLIM separation data, where racemic metoprolol was baseline separated, while racemic panthenol was only partially (∼10%) resolved. Analysis of each separated structure reveals recurring drug structural motifs, namely, bulky cyclic groups and long, flexible carbon chains. These observations are consistent with findings from Yu and Yao on amino acid enantiomer separations.^35^ Large groups, such as benzene, can contribute to larger changes in shape and size (and thus CCS) when the stereocenter is inverted or the molecule is complexed to a selector. Notably, the modeled structure with the lowest *R*_p–p_ was panthenol—the only compound lacking a cyclic group. Additionally, flexible carbon chains in a structure can result in enantiospecific structural differences when they interact with the Cu–His group. This is most prominent in its contribution to the distinct orientations of the metoprolol around Cu–His.

## Conclusions

DTIMS screening of 20 racemic compounds offered insights into copper complex formation as well as narrowing the panel of candidate racemates for HRIM-MS separation of mononuclear histidine complexes. Mononuclear copper complexation using an l-histidine chiral selector resulted in gas-phase enantiospecific structural differences for four drug enantiomers which rendered their corresponding racemic mixtures resolvable by HRIM-MS. Mononuclear [(M)(l-His)(Cu^II^)–H]^+^ complexes were found to be suitable candidates for differentiating enantiomers due to ubiquitous formation and high ion abundance, and unlike higher-order complexes, mononuclear complexes exhibit no ambiguity regarding the chirality of its constituents. TWSLIM-MS analysis of [(M)(l-His)(Cu^II^)–H]^+^ complexes revealed separation of 4 racemic samples and enabled quantitation of thalidomide and metoprolol ee based on peak area ratios. While chiral LC could be used to achieve resolution of almost every chiral drug in our study, here, we note that IM operates on a considerably faster time scale than LC and uses considerably less solvent—thus, adopting IM methods over LC for chiral separations offers both practical and green chemistry benefits. To achieve baseline differentiation of all four racemates that exhibited enantioselective behavior, an *R*_p_ of approximately 550 would be required. This is considerably less than the nearly 2000 *R*_p_ predicted by Dodds et al. to be required for unbound d- and l-leucine,^3^ although whether enantiomers in an achiral environment would exhibit measurable CCS differences is unlikely, but ultimately unknown. To achieve chiral separation using this approach, only 6 ng of drug is required per TWSLIM run. This low sample consumption underscores the utility of this approach in an industrial workflow. Future work will aim to broaden the scope of this analysis workflow by expanding the study to additional chiral selectors (both unmodified and derivatized) as well as additional metal substrates, which may be more selective to different chiral centers. Such broadly targeted screening initiatives will ultimately expand our knowledge of enantioselective IM shifts and allow the application of these strategies to a greater number of drugs and drug-like analytes. Additionally, future work will incorporate LC into the present workflow to achieve quantitation of ee in actual pharmaceutical samples. Ultimately, such strategies may facilitate high-throughput ee measurements in many applications requiring chiral resolution of the analyte species.

## Supporting Information Available

Reagent vendors and structures; instrumental schematics and method information; isotope distributions used in mass spectrum identification; chiral selector evaluation and complex stoichiometry evaluation data; DTIMS racemic drug screening for formation of target complex data; SLIM separation data for mononuclear complexes of racemic drugs; DTIMS and TWSLIM data for unsuccessful separations; thalidomide enantiomeric excess quantitation data; calibration curve data points; calibration curve evaluation and testing; and computational data (PDF)
