# Supplementary material for: Direct Enantiomer Differentiation of Drugs and Drug-Like Compounds via Noncovalent Copper–Amino Acid Complexation and Ion Mobility-Mass Spectrometry
Source: Anal Chem. 2024 Jul 25;96(31):12892–900. doi: 10.1021/acs.analchem.4c02710 (PMC11307251; doi:10.1021/acs.analchem.4c02710)
Supplement: Supplementary file 1 — ac4c02710_si_001.pdf [file ac4c02710_si_001.pdf]

Supplementary Information

Direct Enantiomer Differentiation of Drug and Drug-Like Compounds via Noncovalent Copper-Amino Acid Complexation and Ion Mobility-Mass Spectrometry

Benjamin K. Blakley, Emanuel Zlibut, Rashi M. Gupta, Jody C. May, and John A. McLean*

Department of Chemistry, Center for Innovative Technology, Vanderbilt Institute of Chemical Biology, Vanderbilt-Ingram Cancer Center, and Vanderbilt Institute for Integrated Biosystems Research and Education, Vanderbilt University, Nashville, TN 37235-1822

*Corresponding author email: john.a.mclean@vanderbilt.edu

Comments on Supporting Information Presented in this Work

In this supporting information, we first provide pertinent information for reagents used in this work, including vendors (**Table S1**) and structures (**Figure S1**). We provide schematics for both instruments used throughout the course of the described study (**Figure S2**) and comparisons for TWSLIM-MS spectra obtained using default and optimized method parameters (**Figure S3**). A table containing optimized TWSLIM-MS method parameters is also provided (**Table S2**). In order to facilitate understanding of copper complex identification, theoretical and experimental isotope distributions for mononuclear and binuclear copper complexes of thalidomide and L-histidine are included (**Figure S4**). Results from chiral selector and complex stoichiometry optimization experiments are described in **Text S1**. A full record of measurements made during chiral selector evaluation and copper complex stoichiometry evaluations are described here, including a two-peak resolution ($R_{p\text{-}p}$) heat map organized by complex and chiral selector types (**Figure S5**) and a table summarizing the empirical IM-MS measurements (**Table S3**). Results from the screening of 24 racemic drugs for the target mononuclear complex are described in **Text S2**, and **Table S4** contains corresponding data. Furthermore, DTIMS and TWSLIM spectra for two unsuccessfully separated compounds (i.e., flurbiprofen and chloramphenicol) are provided (**Figure S6**). Next, a table containing mass accuracy, CCS, and abundance values is provided for each racemic drug for which at least one noncovalent copper:histidine complex was observed (**Table S5**). We also provided interday replicate data for each of the four compounds which separated via TWSLIM analysis (**Table S6**). Measurements of *D*-histidine copper complexes are provided in **Table S7**. **Table S8** provides CCS values for each enantiomer. In addition, we include pertinent measurements and calculations for thalidomide and metoprolol enantiomeric excess quantitation in **Tables S9, S10,** and **S11**. The calibration curves themselves are shown in **Figure S8**. Finally, to provide theoretical structural insight to trends in separation, results from computational studies on successfully separated mononuclear complexes are provided in **Figure S9**. To align computational work, ^{DT}CCS values measured in helium drift gas are provided in **Table S12**.

Table of Contents

Figures

Figure No.	Description	Page Number
**S1**	Structures of drug/drug-like compounds and amino acids used in study	S4
**S2**	Instrumental schematics: 6560 and MOBIE	S5
**S3**	Comparison of TWSLIM-MS mass spectra: default vs. optimized	S7
**S4**	Isotope distributions: experimental vs. theoretical	S8
**S5**	$R_{p\text{-}p}$ heat map: chiral selector experiments	S11
**S6**	*D*-histidine measurements: $[(M)(D\text{-His})(Cu^{II}) - H]^+$ profiles	S15
**S7**	PeakLab Fits: Validation of in-house R_P and $R_{P\text{-}P}$ calculations	S19
**S8**	Unsuccessful separation IM spectra	S20
**S9**	Mononuclear $[(M)(L\text{-His})(Cu^{II}) - H]^+$ calibration curve for thalidomide and metoprolol	S21
**S10**	Theoretical work: Conformational space plots and representative average structures for successfully separated mononuclear complexes	S25

Tables

Table No.	Description	Page Number
**S1**	Summary of reagents, vendors, lot IDs, and CAS IDs.	S3
**S2**	Optimized TWSLIM-MS method parameters	S6
**S3**	Comprehensive list of chiral selector evaluation measurements	S10
**S4**	Racemic drug screening results	S13
**S5**	${}^{DT}CCS_{N2}$ and mass accuracy for racemic compounds forming at least one target complex	S14
**S6**	${}^{TWSLIM}CCS_{N2}$ and SLIM separation for racemic compounds forming target complexes; enantiopure measurements for each racemic compound exhibiting separation	S16
**S7**	*D*-histidine measurements: ${}^{TWSLIM}CCS_{N2}$ values	S17
**S8**	TWSLIM data for mononuclear complexes observed on DTIMS platform	S18
**S9**	Interday replicates for mononuclear complexes of racemic compounds exhibiting separation	S22
**S10**	Enantiomeric excess quantitation experiment: measurements, calculation of r, calibration curve data points, and residuals	S23
**S11**	Calibration curve: actual vs. predicted enantiomeric excess, validation using racemic thalidomide and metoprolol	S24
**S12**	Drift tube CCS measurements obtained in helium drift gas (${}^{DT}CCS_{He}$) for successfully separating mononuclear complexes	S26

Texts

Text No.	Description	Page Number
**S1**	Evaluation of Chiral Selectors and Complex Stoichiometries by DTIMS-MS	S9
**S2**	Screening of Racemic Compounds	S12

Table S1. List of reagents used throughout this study, as well as vendors and CAS ID numbers.

Analyte	Source	Lot #/Batch #	CAS #
**Racemic Compounds**			
Penicillamine	Thermo	N25F028	52-66-4
Flurbiprofen	Cayman	102966-58	5104-49-4
Propranolol hydrochloride	Fluka	BCBL2705V	318-98-9
Metoprolol	Fluka	BCBL4001V	56392-17-7
Loratadine	Sigma Aldrich	060M1190V	79794-75-5
Ofloxacin	Cayman	0616767-6	82419-36-1
Salbutamol	Cayman	0494933-31	18559-94-9
Omeprazole	Cayman	0451188-57	13590-58-6
Baclofen	Sigma Aldrich	BCCF9709	1134-47-0
Citalopram	Thermo	A0434754	59729-32-7
Ethosuximide	Sigma Aldrich	LRAA9197	77-67-8
Trimipramine	Sigma Aldrich	080M1613V	52178-8
Thalidomide	Sigma Aldrich	113M4746V	50-35-1
Ibuprofen	Millipore	3738505	15687-27-1
DOPA	Spectrum	2EF0423	63-84-3
Panthenol	TCI	TZYGE-HK	16485-10-2
Alprenolol	EPRS	1.1	13655-52-2
Chloramphenicol	Sigma Aldrich	0000159786	56-75-7
Warfarin	TCI	K3CDM-0P	129-06-6
Atenolol	Fluka	BCBM8864V	29122-68-7
**L-Amino Acids**			
L-Histidine	Sigma Aldrich	BCBG7717V	71-00-1
L-Tryptophan	Sigma Aldrich	113H02901	73-22-3
L-Tyrosine	Sigma Aldrich	91H05604	60-18-4
L-Proline	Sigma Aldrich	128H1156	4305-67-3
**Copper Acetate**			
$Cu(OAc)_2$	Sigma Aldrich	07102ME	142-71-2
**Enantiopure Standards**			
Dexamethasone	Sigma Aldrich	BCBV3213	50-02-2
Betamethasone	Cayman	0491770-2	378-44-9
R-(+)-Thalidomide	Sigma Aldrich	BCBC3292V	2614-06-4
S-(-)-Thalidomide	Sigma Aldrich	061M4628V	841-67-8
R-Ibuprofen	Cayman	0485177-5	51146-57-7
S-Ibuprofen	Sigma Aldrich	BCBR3849V	51146-56-6
R-Metoprolol	Toronto Research Chemical	1-KSS-187-2	81024-43-3
S-Metoprolol	Toronto Research Chemical	1-KSS-187-2	81024-42-2
D-Panthenol	Tokyo Chemical Industry	4E6RG-HM	81-13-0
R-Baclofen	Sigma Aldrich	119K4606	63701-56-4

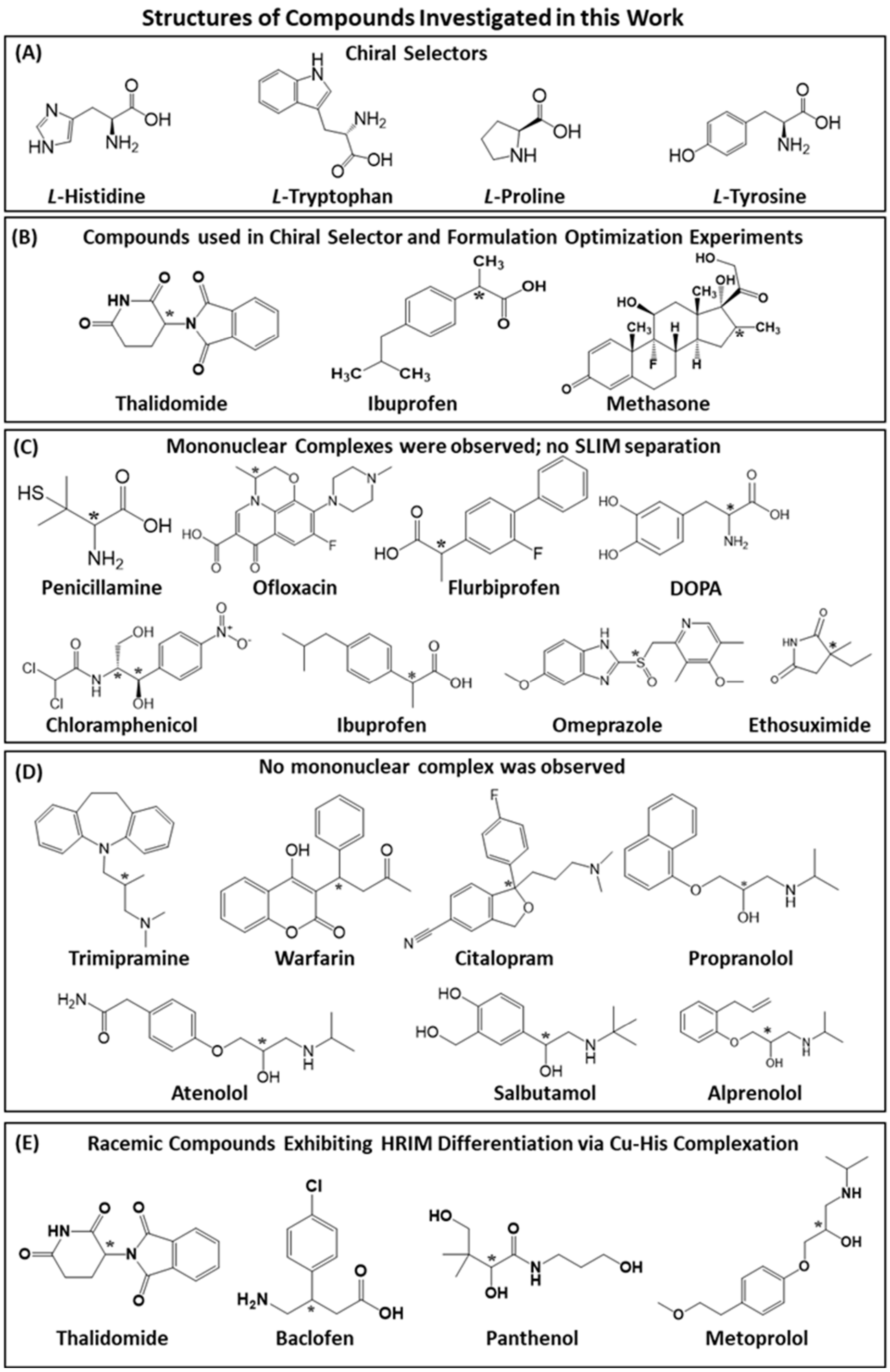

Figure S1. Structures of analyte compounds and amino acids used throughout this work. **(A)** *L*-amino acids explored as chiral selectors. **(B)** Chiral analytes used during chiral selector evaluation. **(C)** Racemic compounds for which mononuclear [(M)(*L*-His)(Cu^{II}) - H]$^+$ complexes were observed, but no separation was observed. **(D)** Racemic compounds for which no mononuclear [(M)(*L*-His)(Cu^{II}) - H]$^+$ complexes were observed on DTIMS instrumentation. **(E)** Racemic compounds which both (1) formed mononuclear copper complexes and (2) exhibited separation on TWSLIM instrumentation. Asterisks indicate positions of chiral centers.

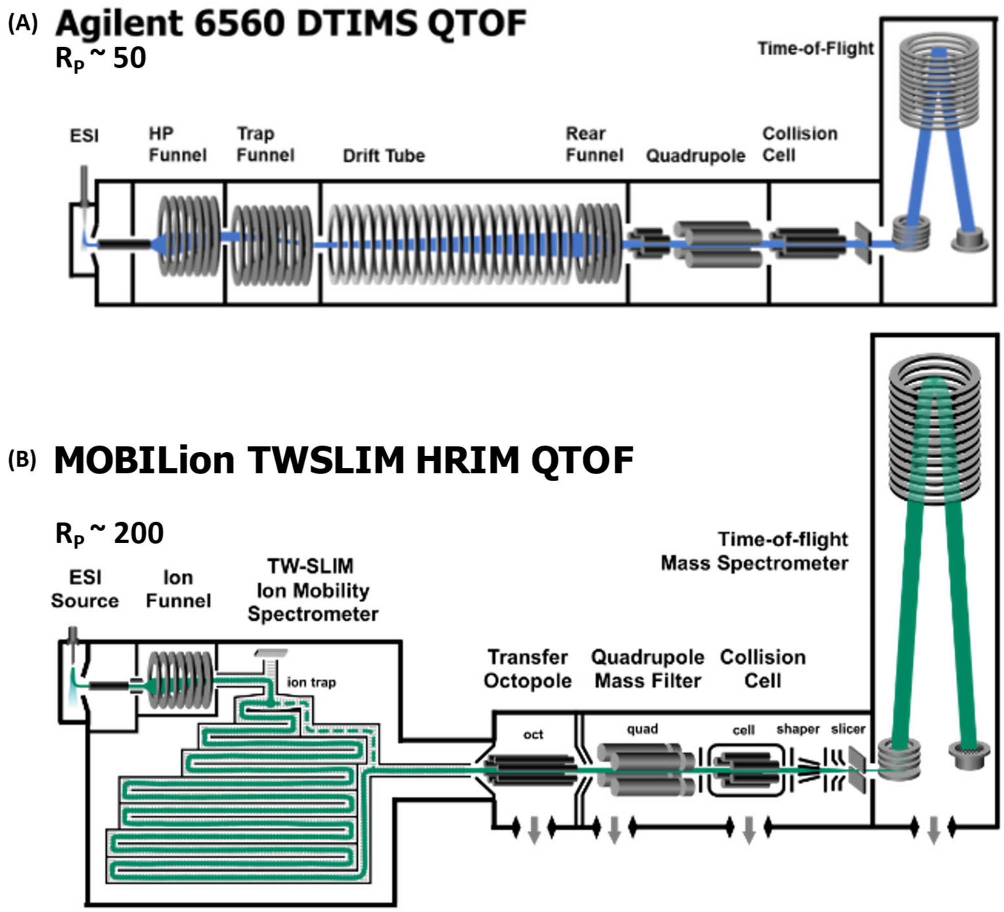

Figure S2. Instrument schematics for the two ion mobility-mass spectrometers used in this study. **(A)** Agilent 6560, a DTIMS-QTOF instrument with a resolving power commonly observed around 50. Based on schematic published in May *et al*. (2015).[1] **(B)** MOBILion MOBIE, a SLIM-based high-resolution TWIMS instrument operating at resolving powers around 200. The TWSLIM is coupled to an Agilent 6546 QTOF. Original artwork by Jody C. May.

(1) May, J. C.; Dodds, J. N.; Kurulugama, R. T.; Stafford, G. C.; Fjeldsted, J. C.; McLean, J. A. Broadscale Resolving Power Performance of a High Precision Uniform Field Ion Mobility-Mass Spectrometer. Analyst 2015, 140 (20), 6824–6833. https://doi.org/10.1039/c5an00923e.

Table S2. List of default and optimized TWSLIM-MS method parameters. Method parameters were optimized relative to the abundance of mononuclear copper complexes. Bold values in the optimize column were modified during method optimization.

Instrument Parameter	Default	Optimized
Fragmentor (V)	165	**125**
Skimmer (V)	45	45
Oct 1 RF Vpp (V)	750	750
Oct 1 DC (V)	28.1	**25.3**
Lens 1 (V)	26.5	**23.8**
Lens 2 (V)	18.1	**9.1**
Lens 2 RF V (V)	0	0
Lens 2 RF Ph (deg)	54	54
Quad AMU (amu)	100	**117.3**
Quad DC (V)	24.9	**21.8**
PostFilter DC (V)	23.6	**21.5**
Width Gain	1744	1744
Width Offset	1481	1481
Axis Gain	1245	1245
Axis Offset	2541	2541
Cell Entrance (V)	21.4	**20.5**
Hex RF (V)	550	550
Hex DC (V)	21.3	**19.8**
Hex Delta (V)	-7	**-2**
Hex2 DC (V)	14.3	**17.8**
Hex2 RF (V)	600	600
Hex2 DV (V)	-1	-1
Hex3 DC (V)	13.1	**15.5**
Ion Focus (V)	9.4	**13.3**
Extractor DC (V)	-10	**-9.7**
Top Slit (V)	-40.1	**-40.3**
Bot Slit (V)	-39.6	-39.6
Lens 3 (V)	-59.3	**-56.3**
Pusher Offset (mV)	-76	-76
Pusher (V)	1200	1200
Puller (V)	-700	-700
Puller Offset (V)	34.88	34.88
Acc Focus (V)	-2146	**-2064**
Mirror Front (V)	-9000	-9000
Mirror Mid (V)	-2273.9	**-2273.6**
Mirror Back (V)	1475	1475
Min Mass Range (m/z)	50	**20**
Max Mass Range (m/z)	1700	1700
Acquisition Rate (spectra/s)	1	1
Time (ms/spectrum)	1000	1000
MCP (V)	693	693
Amplifier Offset (DAC)	2747	2747
Separation TW Amplitude (V)	40	**20**
Separation TW Frequency (kHz)	20	**10**

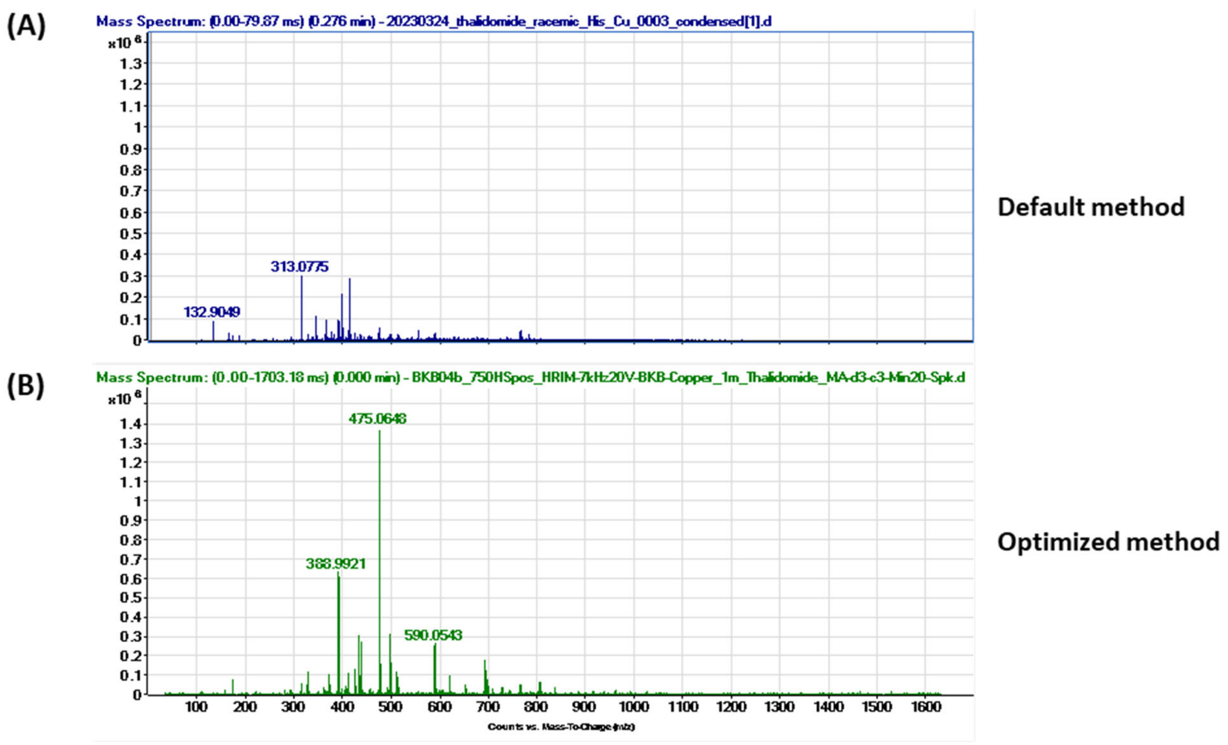

Figure S3. TWSLIM-MS mass spectrum using **(A)** default method and **(B)** optimized method. Method optimization was performed to promote transmission of mononuclear complexes (i.e., *m/z* 475.0648 in optimized panel).

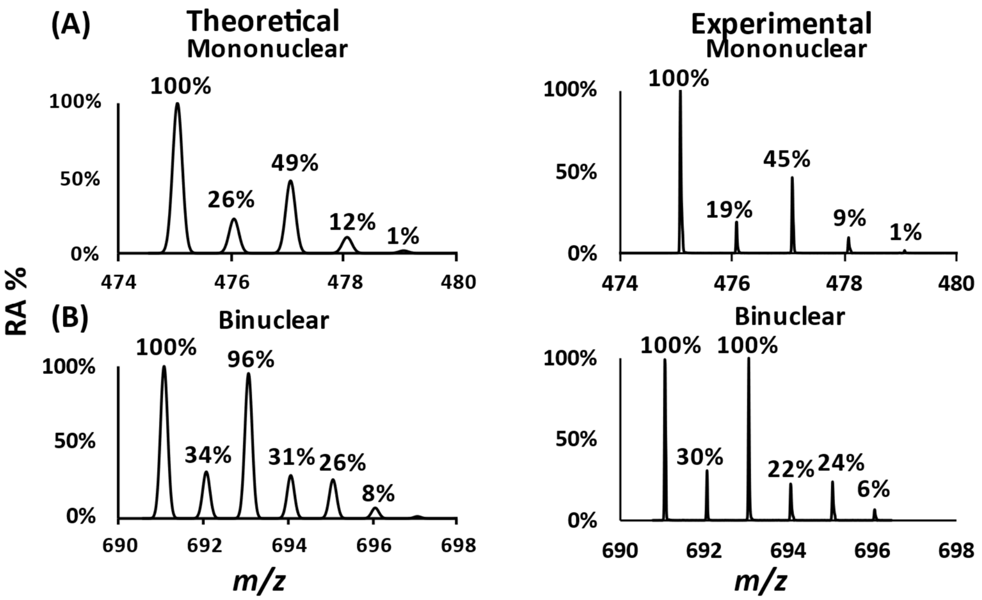

Figure S4. Theoretical and experimental isotope distributions for **(A)** mononuclear [(THA)(*L*-His)(Cu^{II}) − H]$^+$ and **(B)** binuclear [(THA)(*L*-His)$_2$(Cu^{II})$_2$ − 3H]$^+$ copper complexes. To facilitate comparison, isotope abundance percentages (relative to base beak in isotopic envelope) are provided above each peak. (RA = Relative ion abundance)

Evaluation of Chiral Selectors and Complex Stoichiometries by DTIMS-MS. Similar stoichiometries were observed among the various analyte and chiral amino acids evaluated. In addition to high MS abundance, stoichiometries incorporating only a single analyte yields IM spectra which are much more straightforward to interpret than higher order complexes, specifically because single-analyte complexes avoid the possibility of heterochirality. This is in contrast to higher-order complexes where the incorporation of additional analytes can result in mixed chirality complexes (i.e., heterochiral *R* + *S* vs. homochiral *R* + *R* and *S* + *S*) which can complicate IM spectral interpretation.

Further studies were designed to evaluate copper complexes incorporating various amino acid chiral selectors. A panel of three chiral drugs (Dexa/Betamethasone, *R/S*-ibuprofen, and *R/S*-thalidomide) were used to evaluate four aromatic amino acids as chiral selectors (*L*-histidine, *L*-tryptophan, *L*-tyrosine, and *L*-proline). These three drugs were selected for this evaluation due to ease of access to their enantiopure forms at the time of experimental design. Mononuclear $[(M)(AA)(Cu^{II}) - H]^+$ and $[(M)(AA)(Cu^{II}) + Na - 2H]^+$ as well as binuclear $[(M)(AA)_2(Cu^{II})_2 - 3H]^+$ stoichiometries were considered, where M represents the analyte. $R_{p\text{-}p}$ was calculated for each drug pair at each target complex stoichiometry using $^{DT}CCS_{N2}$ values obtained from each enantiopure IM-MS spectrum. **Table S3** contains a comprehensive list of $^{DT}CCS_{N2}$ and $R_{p\text{-}p}$ values measured for each observed M:CS:Cu complex, and **Figure S5** summarizes the measured $R_{p\text{-}p}$ values as a heatmap organized by complex stoichiometry, chiral selector type, and analyte.

While *L*-tyrosine and *L*-tryptophan achieved nonzero two-peak resolutions for sodiated mononuclear Dexa/Betamethasone ($R_{p\text{-}p}$ of 0.45 and 0.32, respectively), neither selector resulted in consistent observation of target complexes. Similar results were observed for *L*-proline, which exhibited an $R_{p\text{-}p}$ of 0.32 for sodiated mononuclear *R/S*-ibuprofen, but not all target complexes were observed for this analyte. However, *L*-histidine (blue dashed box, **Figure S5**) demonstrated both formation of all target complexes and measurable differentiation of the racemate enantiomer pairs. Notably, an $R_{p\text{-}p}$ of 0.58 (~10% separation) was achieved for mononuclear [(Dexa/Betamethasone)(*L*-His)$(Cu^{II}) - H]^+$. This is a somewhat intuitive result, as histidine, a basic aromatic residue, is linked to improved cation-π interactions[1] as well as known chiral differentiation when bound to divalent copper.[2] Because of histidine's propensity to form enantioselective noncovalent complexes, it was selected as the amino acid CS for future experiments.

References

(1) Pirkle, W. H.; Pochapsky, T. C. Considerations of Chiral Recognition Relevant to the Liquid Chromatographic Separation of Enantiomers. Chem. Rev. 1989, 89, 347–362. https://doi.org/10.1021/cr00092a006.

(2) Zlibut, E.; May, J. C.; Mclean, J. A. Enantiomer Differentiation of Amino Acid Stereoisomers by Structural Mass Spectrometry Using Noncovalent Trinuclear Copper Complexes. J. Am. Soc. Mass Spectrom. 2022, 33, 58. https://doi.org/10.1021/jasms.2c00059.

Table S3. Mass accuracy, CCS, and two-peak resolution (R_{p-p}) values obtained from chiral selector evaluation and complex stoichiometry evaluation experiments. Complex types under evaluation include $[(M)(AA)(Cu^{II}) – H]^+$, $[(M)(AA)(Cu^{II}) + Na – 2H]^+$, and $[(M)(AA)_2(Cu^{II})_2 – 3H]^+$. Compounds not observed in IM-MS spectrum are not included in the table.

AA	Complex type	Analyte (M)	Form	Theoretical Mass [Da]	Mass Accuracy [ppm]	$^{DT}CCS_{N2}$ [Å^2]	R_{p-p}
**Tryptophan**	[M + AA + Cu + Na - 2H]+	Methasone	*Dexa*	680.1935	3.2 ± 3.9	232.36 ± 0.56	0.32 ± 0.05
	[M + AA + Cu + Na - 2H]+	Methasone	*Beta*	680.1935	-4.9 ± 3.3	229.73 ± 0.37	
	[M + 2AA + 2Cu - 3H]+	Methasone	*Dexa*	923.2154	7.6 ± 3.1	261.36 ± 0.43	0.08 ± 0.01
	[M + 2AA + 2Cu - 3H]+	Methasone	*Beta*	923.2154	-1.8 ± 4.8	260.52 ± 0.62	
	[M + AA + Cu + Na - 2H]+	Thalidomide	*R*	546.0576	4.4 ± 1.2	207.61 ± 0.30	0.31 ± 0.01
	[M + AA + Cu + Na - 2H]+	Thalidomide	*S*	546.0576	-1.9 ± 5.0	205.56 ± 0.33	
**Proline**	[M + AA + Cu - H]+	Methasone	*Dexa*	570.1928	1.1 ± 4.5	234.78 ± 0.39	0.07 ± 0.02
	[M + AA + Cu - H]+	Methasone	*Beta*	570.1928	1.8 ± 5.6	234.84 ± 0.24	
	[M + AA + Cu + Na - 2H]+	Ibuprofen	*R*	405.0977	16.1 ± 9.0	191.75 ± 6.95	0.32 ± 0.2
	[M + AA + Cu + Na - 2H]+	Ibuprofen	*S*	405.0977	15.8 ± 5.7	195.00 ± 6.72	
	[M + AA + Cu + Na - 2H]+	Thalidomide	*R*	457.0311	-1.0 ± 4.5	199.03 ± 0.31	0.06 ± 0.03
	[M + AA + Cu + Na - 2H]+	Thalidomide	*S*	457.0311	-1.5 ± 6.3	198.84 ± 0.42	
**Tyrosine**	[M + AA + Cu + Na - 2H]+	Methasone	*Dexa*	657.1775	-3.4 ± 7.4	227.25 ± 0.78	0.45 ± 0.2
	[M + AA + Cu + Na - 2H]+	Methasone	*Beta*	657.1775	-5.7 ± 5.1	230.26 ± 0.68	
	[M + 2AA + 2Cu - 3H]+	Methasone	*Dexa*	877.1834	-4.8 ± 7.6	252.54 ± 0.24	0.28 ± 0.01
	[M + 2AA + 2Cu - 3H]+	Methasone	*Beta*	877.1834	-4.2 ± 8.0	250.21 ± 0.44	
	[M + AA + Cu + Na - 2H]+	Ibuprofen	*R*	471.1083	5.8 ± 5.6	212.06 ± 2.85	0.02 ± 0.01
	[M+ AA + Cu + Na - 2H]+	Ibuprofen	*S*	471.1083	-0.7 ± 8.3	212.17 ± 2.96	
	[M + AA + Cu + Na - 2H]+	Thalidomide	*R*	523.0417	0.4 ± 5.4	204.71 ± 0.30	0.01 ± 0.01
	[M + AA + Cu + Na - 2H]+	Thalidomide	*S*	523.0417	-3.9 ± 5.3	204.79 ± 0.33	
**Histidine**	[M + AA + Cu - H]+	Methasone	*Dexa*	609.1911	3.7 ± 0.6	224.48 ± 1.42	0.58 ± 0.06
	[M + AA + Cu - H]+	Methasone	*Beta*	609.1911	2.9 ± 0.1	223.10 ± 1.73	
	[M + AA + Cu + Na - 2H]+	Methasone	*Dexa*	631.1731	4.5 ± 2.8	224.91 ± 0.56	~0.00
	[M + AA + Cu + Na - 2H]+	Methasone	*Beta*	631.1731	1.9 ± 0.4	224.92 ± 0.61	
	[M + 2AA + 2Cu - 3H]+	Methasone	*Dexa*	825.1746	3.6 ± 1.7	249.95 ± 1.01	0.18 ± 0.06
	[M + 2AA + 2Cu - 3H]+	Methasone	*Beta*	825.1746	1.8 ± 0.3	252.67 ± 1.13	
	[M + AA + Cu - H]+	Ibuprofen	*R*	423.1219	-0.8 ± 1.1	204.24 ± 0.15	0.03 ± 0.02
	[M + AA + Cu - H]+	Ibuprofen	*S*	423.1219	-2.2 ± 0.6	203.50 ± 1.13	
	[M + AA + Cu + Na - 2H]+	Ibuprofen	*R*	445.1039	-2.0 ± 1.9	195.47 ± 0.51	0.01 ± 0.01
	[M + AA + Cu + Na - 2H]+	Ibuprofen	*S*	445.1039	-2.8 ± 1.5	195.53 ± 0.51	
	[M + 2AA + 2Cu - 3H]+	Ibuprofen	*R*	639.1053	2.4 ± 2.8	225.70 ± 0.40	0.02 ± 0.01
	[M + 2AA + 2Cu - 3H]+	Ibuprofen	*S*	639.1053	5.1 ± 3.3	225.80 ± 0.41	
	[M + AA + Cu - H]+	Thalidomide	*R*	475.0553	2.4 ± 4.7	203.53 ± 0.59	0.21 ± 0.03
	[M + AA + Cu - H]+	Thalidomide	*S*	475.0553	4.4 ± 3.1	203.31 ± 0.34	
	[M + AA + Cu + Na - 2H]+	Thalidomide	*R*	497.0372	3.9 ± 4.7	199.17 ± 0.70	0.18 ± 0.04
	[M + AA + Cu + Na - 2H]+	Thalidomide	*S*	497.0372	5.7 ± 3.9	200.27 ± 0.48	
	[M + 2AA + 2Cu - 3H]+	Thalidomide	*R*	691.0387	3.4 ± 4.5	231.66 ± 0.40	0.31 ± 0.03
	[M + 2AA + 2Cu - 3H]+	Thalidomide	*S*	691.0387	4.6 ± 3.2	233.91 ± 0.64	

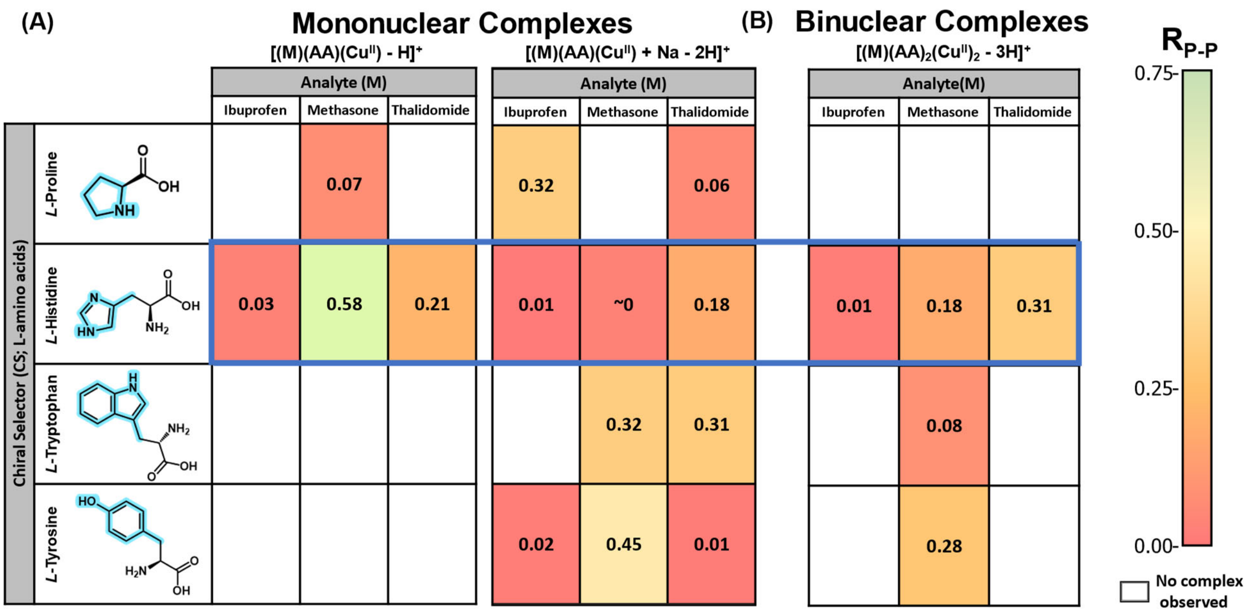

Figure S5. Heat maps summarizing two-peak resolutions ($R_{p\text{-}p}$) found during chiral selector (CS) and formulation optimization experiments for three candidate complexes: **(A)** mononuclear $[(M)(AA)(Cu^{II}) - H]^+$ and mononuclear $[(M)(AA)(Cu^{II}) + Na - 2H]^+$, and **(B)** binuclear $[(M)(AA)_2(Cu^{II})_2 - 3H]^+$. Compounds explored during these experiments include *R*/*S*-ibuprofen, Dexa/Betamethasone, and *R*/*S*-thalidomide. Chiral selectors explored include *L*-proline, *L*-histidine, *L*-tryptophan, and *L*-tyrosine. Notably, all three target complexes were observed for all three analyte compounds when *L*-histidine was used as the chiral selector.

Screening of Racemic Compounds. Twenty racemic compounds were subsequently screened with Cu-His via DTIMS. For each racemate screened, formation of the mononuclear $[(M)(L\text{-His})(Cu^{II}) - H]^+$ complex was of primary interest. Additional screening targets included four Cu:His complexes not bound to M, as well as the sodium-coordinated mononuclear $[(M)(L\text{-His})(Cu^{II}) + Na - 2H]^+$ and binuclear $[(M)(L\text{-His})_2(Cu^{II})_2 - 3H]^+$ complexes. **Table S4** summarizes the full list of racemic compounds, target complexes, and approximate abundances observed for each complex. All four target unbound His:Cu complexes were observed for all twenty racemic compounds, while thirteen of the twenty racemates (65%) formed at least one mononuclear complex stoichiometry. The binuclear $[(M)(L\text{-His})_2(Cu^{II})_2 - 3H]^+$ complex was only observed in a few of the racemate systems and only when the mononuclear complex was also observed. Abundances and $^{DT}CCS_{N2}$ measurements corresponding to each target complex observed are summarized in **Table S5**.

Interestingly, the screening results provide interesting insight into the formation of M:His:Cu complexes. Specifically, no higher order complex was observed without also observing the lower order complex(es), and the analyte-incorporated complexes were only observed when the analyte-excluded AA:Cu complex appeared in high abundance. These observations suggest that a histidine ligand must first coordinate with copper, and then the chiral analyte interacts with this binary Cu:His core complex to form the ternary mononuclear $[(M)(L\text{-His})(Cu^{II}) - H]^+$ complex. This proposed formation scheme is consistent with published DFT work on histidine-copper complex stability[3] as well as chiral selector-analyte interactions.[1,4] In order to form the higher-order binuclear $[(M)(L\text{-His})_2(Cu^{II})_2 - 3H]^+$ complex, we infer that a second Cu:His coordination complex combines with a mononuclear complex. As a result, higher-order complexes (binuclear, trinuclear, etc.) can only form when conditions are favorable for the formation of a mononuclear complex.

References

(1) Pirkle, W. H.; Pochapsky, T. C. Considerations of Chiral Recognition Relevant to the Liquid Chromatographic Separation of Enantiomers. Chem. Rev. 1989, 89, 347–362. https://doi.org/10.1021/cr00092a006.

(2) Zlibut, E.; May, J. C.; Mclean, J. A. Enantiomer Differentiation of Amino Acid Stereoisomers by Structural Mass Spectrometry Using Noncovalent Trinuclear Copper Complexes. J. Am. Soc. Mass Spectrom. 2022, 33, 58. https://doi.org/10.1021/jasms.2c00059.

(3) Remko, M.; Fitz, D.; Rode, B. M. Effect of Metal Ions (Li+, Na+, K+, Mg2+, Ca2+, Ni2+, Cu2+ and Zn2+) and Water Coordination on the Structure and Properties of l-Histidine and Zwitterionic l-Histidine. Amino Acids 2010, 39 (5), 1309–1319. https://doi.org/10.1007/s00726-010-0573-8.

(4) Berthod, A. Chiral Recognition Mechanisms. Anal. Chem. 2006, 78 (7), 2093–2099. https://doi.org/10.1021/ac0693823.

Table S4. Tabular summary of racemic compound screening. Values in the table represent absolute abundances, and here we note that these values are indicative of orders of magnitude across interday replicates.

			AA:Cu				M:AA:Cu			
			1:1	2:2	3:2	5:3	1:1:1	1:1:1	1:2:2	3:2:3
	Analyte (M)	AA	$[(AA)(Cu) - CH_2O_2]^+$	$[(AA)_2(Cu)_2 - CH_3O_2]^+$	$[(AA)_3(Cu)_2 - 3H]^+$	$[(AA)_5(Cu)_3 - 5H]^+$	$[(M)(AA)(Cu) - H]^+$	$[(M)(AA)(Cu) + Na - 2H]^+$	$[(M)(AA)_2(Cu)_2 - 3H]^+$	$[(M)_3(AA)_2(Cu)_3 - 5H]^+$
Formed mononuclear complex	*Baclofen*	His	1E4	2E5	1E4	2E3	1E4	1E4	6E4	1E3
	Chloramphenicol	His	2E4	2E5	2E4	9E3	9E3			
	DOPA	His	8E4	2E5	5E4	4E3	4E3	2E4		
	Ethosuximide	His	1E4	2E5	2E4	8E3	8E3	8E2	3E3	
	Flurbiprofen	His	1E5	1E6	2E5	3E4	3E4	8E4	2E5	
	Metoprolol	His	7E4	6E5	1E5	3E4	5E4	2E4	2E3	
	Panthenol	His	4E4	3E5	7E4	5E4	1E4	1E4		
	Penicillamine	His	8E4	2E5	5E4	1E4	5E3		2E3	
	Ofloxacin	His	1E5	9E5	4E4	5E3	1E7	7E5	1E5	
	Omeprazole	His	3E4	7E5	2E5	7E4	4E4	3E4		
	Thalidomide	His	1E5	8E5	1E5	3E4	4E5	5E4	2E4	
		Pro	1E3	5E4	7E4	8E4		3E5		
		Tyr	9E4	1E5	3E4	1E5		1E6		
		Trp	3E5	7E5	2E4	7E5		4E5		
	Methasone	His	8E4	1E6	5E4	4E4	3E4	3E4	1E3	
		Pro	1E3	9E3	4E4	4E4	4E3			
		Tyr	7E4	8E4	5E3	1E5		5E4	1E5	
		Trp	2E5	5E5	1E4	2E5		1E5	1E5	
	Ibuprofen	His	1E5	2E6	2E5	7E4	6E4	1E5	1E6	
		Pro	1E3	1E4	1E5	1E5		1E4		
		Tyr	1E5	5E4	1E4	7E4		6E4		
		Trp	1E5	8E5	8E3	1E5		9E4		
No mononuclear complex	*Salbutamol*	His	8E4	6E5	1E5	5E3				
	Propranolol	His	6E4	7E5	1E5	3E4				
	Alprenolol	His	3E3	1E4	2E3	7E2				
	Atenolol	His	4E3	2E4	3E3	3E3				
	Citalopram	His	3E3	9E4	8E3	1E3				
	Trimipramine	His	2E3	5E4	5E4	2E2				
	Warfarin	His	6E3	1E5	1E4	2E3				

MS Abundance *<E2* *E2* *E3* *E4* *E5* *>E6*

Table S5. Abundance, mass accuracy, and $^{DT}CCS_{N2}$ values measured for racemic compounds for which at least one target copper complex was observed. For compound type, "mono" refers to mononuclear complexes (that is, one copper in the complex), and "bi" refers to binuclear complexes (two coppers in the complex. "Average abundance" refers to the average of three interday measurements of MS intensity for a given complex.

Analyte (M)	Complex	Type	Theoretical Mass (Da)	Average Abundance	Mass Accuracy (ppm)	$^{DT}CCS_{N2}$ ± SD (Å^2)	RSD (%)
**Thalidomide**	$[(M)(L\text{-His})(Cu^{II}) - H]^+$	Mono	475.0553	184,357	-6.1 ± 0.5	202.72 ± 0.20	0.20
	$[(M)(L\text{-His})(Cu^{II}) + Na - 2H]^+$	Mono	497.0372	34,865	-18.5 ± 4.9	199.33 ± 0.13	0.12
	$[(M)(L\text{-His})_2(Cu^{II})_2 - 3H]^+$	Bi	691.0387	11,040	-6.1 ± 1.7	232.64 ± 0.09	0.07
**DOPA**	$[(M)(L\text{-His})(Cu^{II}) - H]^+$	Mono	414.0601	3,012	-12.7 ± 5.0	180.51 ± 0.30	0.29
	$[(M)(L\text{-His})(Cu^{II}) + Na - 2H]^+$	Mono	436.0419	12,030	-8.5 ± 1.2	185.00 ± 0.18	0.17
	$[(M)(L\text{-His})_2(Cu^{II})_2 - 3H]^+$	Bi	630.0435	not observed			
**Panthenol**	$[(M)(L\text{-His})(Cu^{II}) - H]^+$	Mono	422.1226	29,784	-7.1 ± 0.5	189.34 ± 0.12	0.11
	$[(M)(L\text{-His})(Cu^{II}) + Na - 2H]^+$	Mono	444.1046	17,915	-8.7 ± 0.1	191.92 ± 0.12	0.11
	$[(M)(L\text{-His})_2(Cu^{II})_2 - 3H]^+$	Bi	638.1061	not observed			
**Penicillamine**	$[(M)(L\text{-His})(Cu^{II}) - H]^+$	Mono	366.0422	6,395	-5.9 ± 0.8	173.53 ± 0.06	0.06
	$[(M)(L\text{-His})(Cu^{II}) + Na - 2H]^+$	Mono	388.0242	not observed			
	$[(M)(L\text{-His})_2(Cu^{II})_2 - 3H]^+$	Bi	582.02572	1,082	2.1 ± 9.4	203.52 ± 0.38	0.32
**Flurbiprofen**	$[(M)(L\text{-His})(Cu^{II}) - H]^+$	Mono	461.0812	76,204	-5.4 ± 0.5	211.28 ± 0.18	0.15
	$[(M)(L\text{-His})(Cu^{II}) + Na - 2H]^+$	Mono	483.0631	41,183	-6.6 ± 0.4	201.18 ± 0.18	0.17
	$[(M)(L\text{-His})_2(Cu^{II})_2 - 3H]^+$	Bi	677.0646	98,511	-3.3 ± 0.1	232.04 ± 0.19	0.14
**Metoprolol**	$[(M)(L\text{-His})(Cu^{II}) - H]^+$	Mono	484.1747	5,531	-10.6 ± 3.5	196.81 ± 0.29	0.26
	$[(M)(L\text{-His})(Cu^{II}) + Na - 2H]^+$	Mono	506.1566	19,265	-8.3 ± 1.7	200.38 ± 0.20	0.17
	$[(M)(L\text{-His})_2(Cu^{II})_2 - 3H]^+$	Bi	700.1581	2,202	-26.3 ± 7.4	235.83 ± 0.88	0.64
**Ofloxacin**	$[(M)(L\text{-His})(Cu^{II}) - H]^+$	Mono	578.1350	11,477,065	7.7 ± 2.9	236.58 ± 0.12	0.09
	$[(M)(L\text{-His})(Cu^{II}) + Na - 2H]^+$	Mono	600.1169	745,903	-3.3 ± 2.4	237.18 ± 0.16	0.12
	$[(M)(L\text{-His})_2(Cu^{II})_2 - 3H]^+$	Bi	794.1184	147,719	-7.2 ± 1.1	263.60 ± 0.37	0.24
**Baclofen**	$[(M)(L\text{-His})(Cu^{II}) - H]^+$	Mono	430.0469	21,922	-9.1 ± 0.3	189.50 ± 0.07	0.07
	$[(M)(L\text{-His})(Cu^{II}) + Na - 2H]^+$	Mono	452.0288	25,398	-7.9 ± 0.4	192.77 ± 0.13	0.11
	$[(M)(L\text{-His})_2(Cu^{II})_2 - 3H]^+$	Bi	646.0303	98,775	-5.1 ± 0.5	219.28 ± 0.10	0.08
**Chloramphenicol**	$[(M)(L\text{-His})(Cu^{II}) - H]^+$	Mono	539.0035	5,121	-12.4 ± 1.0	211.48 ± 0.19	0.16
	$[(M)(L\text{-His})(Cu^{II}) + Na - 2H]^+$	Mono	560.9855	not observed			
	$[(M)(L\text{-His})_2(Cu^{II})_2 - 3H]^+$	Bi	754.9869	not observed			
**Ethosuximide**	$[(M)(L\text{-His})(Cu^{II}) - H]^+$	Mono	358.0702	1,318	-0.5 ± 1.2	175.40 ± 0.56	0.55
	$[(M)(L\text{-His})(Cu^{II}) + Na - 2H]^+$	Mono	380.0521	not observed			
	$[(M)(L\text{-His})_2(Cu^{II})_2 - 3H]^+$	Bi	574.0536	5,661	-2.8 ± 0.9	205.97 ± 0.17	0.14
**Omeprazole**	$[(M)(L\text{-His})(Cu^{II}) - H]^+$	Mono	562.1059	43,244	-3.1 ± 4.0	220.43 ± 1.79	1.41
	$[(M)(L\text{-His})(Cu^{II}) + Na - 2H]^+$	Mono	584.0878	37,731	-8.7 ± 2.2	221.53 ± 1.64	1.29
	$[(M)(L\text{-His})_2(Cu^{II})_2 - 3H]^+$	Bi	778.0893	not observed			
**Ibuprofen**	$[(M)(L\text{-His})(Cu^{II}) - H]+$	Mono	423.1219	5,648,756	-0.4 ± 2.0	203.64 ± 0.07	0.058
	$[(M)(L\text{-His})(Cu^{II}) + Na - 2H]+$	Mono	497.0372	335,087	-4.5 ± 2.2	194.71 ± 0.07	0.065
	$[(M)(L\text{-His})_2(Cu^{II})_2 - 3H]+$	Bi	691.0387	6,025,602	0.04 ± 2.1	225.00 ± 0.07	0.066

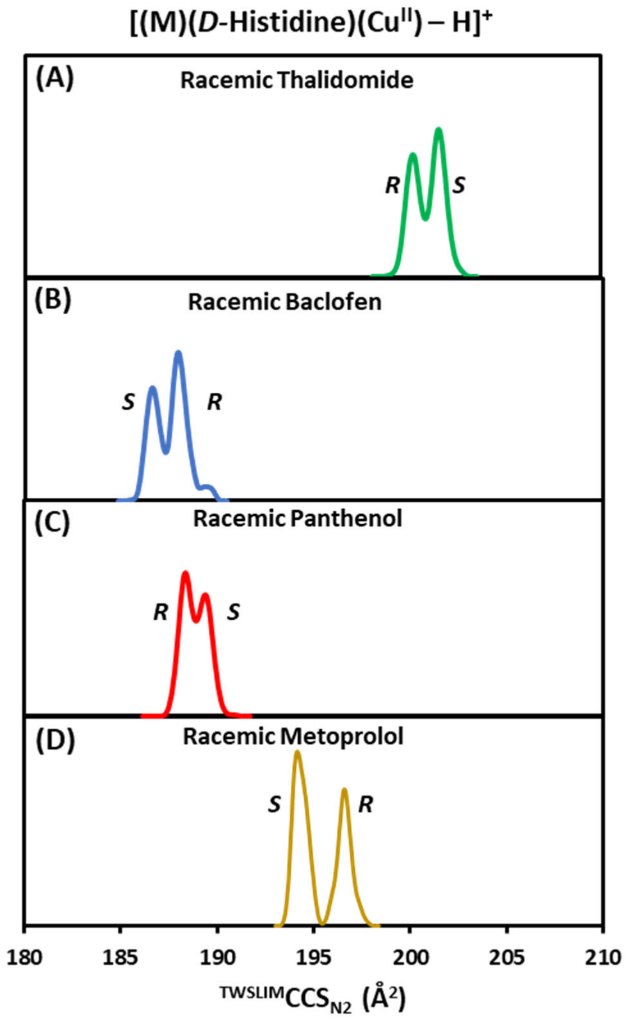

Figure S6. TWSLIM profiles for mononuclear [(M)(*D*-Histidine)(Cu^{II}) – H]$^+$. Notably, incorporation of *D*-histidine into ternary copper complex resulted in inversion of drug enantiomer arrival order, but the profiles were identical.

Table S6. Interday reproducibility data for racemic compounds whose mononuclear [(M)(*L*-His)(Cu^{II}) – H]$^+$ complex exhibited TWSLIM-MS differentiation. To illustrate how peak identifications were made within each racemic TWSLIM spectrum, measurements for commerically available enantiopure drug standards are included. Because *L*-panthenol and *S*-baclofen were not commercially available, their measurements are not included. Identifications were made based on alignment of enantiopure CCS with the CCS of a given racemic TWSLIM peak.

L-Histidine Results

Racemic Samples: [(M)(^{L}His)(Cu^{II}) – H]$^+$							
**Analyte (M)**	**$^{TWSLIM}CCS_{N2}$ (Å^2)**		**$\Delta^{TWSLIM}CCS_{N2}$ (Å^2)**	**ΔCCS%**	**R_{p-p}**	**Average R_{p-p}**	**Standard Error**
	**Enantiomer 1**	**Enantiomer 2**					
**Thalidomide**	200.51	201.86	1.35	0.67%	0.83	0.87	0.03
	199.81	201.14	1.33	0.66%	0.89		
	199.85	201.23	1.38	0.69%	0.88		
**Panthenol**	188.57	189.57	1.00	0.53%	0.68	0.72	0.05
	187.80	188.78	0.98	0.52%	0.70		
	187.82	188.86	1.04	0.55%	0.79		
**Metoprolol**	194.58	196.88	2.30	1.18%	1.47	1.52	0.06
	193.83	196.13	2.30	1.18%	1.57		
	193.89	196.14	2.25	1.15%	1.53		
**Baclofen**	186.77	188.15	1.38	0.74%	0.88	0.89	0.01
	186.01	187.36	1.35	0.72%	0.90		
	186.07	187.43	1.36	0.73%	0.90		

Example:

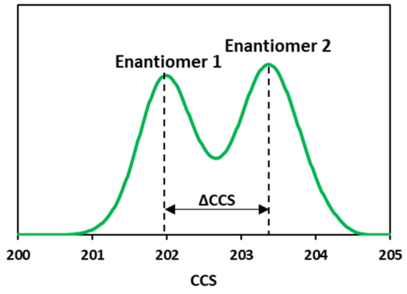

Enantiopure Samples: [(M)(*L*-His)(Cu^{II}) – H]$^+$		
**Analyte (M)**	**$^{TWSLIM}CCS_{N2}$ (Å^2)**	**Corresponds to…**
S-Thalidomide	199.33	Enantiomer 1
R-Thalidomide	199.62	Enantiomer 2
R-Metoprolol	194.48	Enantiomer 1
S-Metoprolol	196.76	Enantiomer 2
D-Panthenol	189.48	Enantiomer 2
R-Baclofen	186.68	Enantiomer 1

Table S7. $^{TWSLIM}CCS_{N2}$ measurements for mononuclear [(M)(*D*-Histidine)(Cu^{II}) – H]$^+$ complexes. *D*-histidine measurements align well to *L*-histidine measurements. Enantiopure measurements confirm that changing the chirality of histidine results in inversion of copper complex arrival order.

D-Histidine Results

Racemic Samples: [(M)(*D*-His)(Cu^{II}) – H]$^+$			
**Analyte (M)**	**$^{TWSLIM}CCS_{N2}$ (Å^2)**		**$\Delta^{TWSLIM}CCS_{N2}$ (Å^2)**
	**Enantiomer 1**	**Enantiomer 2**	
**Thalidomide**	200.28	201.53	1.25
**Panthenol**	188.32	189.37	1.05
**Metoprolol**	194.00	196.35	2.35
**Baclofen**	186.51	187.88	1.37

Enantiopure Samples: [(M)(*D*-His)(Cu^{II}) – H]$^+$		
**Analyte (M)**	**$^{TWSLIM}CCS_{N2}$ (Å^2)**	**Corresponds to…**
S-Thalidomide	201.53	Enantiomer 2
R-Thalidomide	200.28	Enantiomer 1
R-Metoprolol	196.64	Enantiomer 2
S-Metoprolol	194.35	Enantiomer 1
D-Panthenol	188.35	Enantiomer 1
R-Baclofen	187.97	Enantiomer 2

Table S8. TWSLIM-MS separation data (with DTIMS CCS data included for comparison) for each racemic compounds with an observed DTIMS mononuclear copper complex. CCS data is only included for complexes where some amount of separation ($R_{p\text{-}p} > 0$) was observed.

Analyte (M)	Complex	$^{DT}CCS_{N2}$ (Å^2)	SLIM Sep?	$^{TWSLIM}CCS_{N2}$ (Å^2)		$\Delta^{TWSLIM}CCS_{N2}$ (Å^2)	ΔCCS%	$R_{p\text{-}p}$
**Thalidomide**	**[(M)(*L*-His)(Cu^{II}) - H]$^+$**	202.72 ± 0.23	Y	200.51	201.86	1.35	0.67%	0.87
	**[(M)(*L*-His)(Cu^{II}) + Na - 2H]$^+$**	199.33 ± 0.13	N					
	**[(M)(*L*-His)$_2$(Cu^{II})$_2$ - 3H]$^+$**	232.64 ± 0.09	Y	229.14	231.75	2.61	1.13%	1.51
**DOPA**	**[(M)(*L*-His)(Cu^{II}) - H]$^+$**	180.51 ± 0.30	N					
	**[(M)(*L*-His)(Cu^{II}) + Na - 2H]$^+$**	185.02 ± 0.18	N					
	**[(M)(*L*-His)$_2$(Cu^{II})$_2$ - 3H]$^+$**	Not observed in DTIMS screening; not searched in TWSLIM spectrum.						
**Panthenol**	**[(M)(*L*-His)(Cu^{II}) - H]$^+$**	189.34 ± 0.12	Y	188.57	189.57	1.00	0.53%	0.72
	**[(M)(*L*-His)(Cu^{II}) + Na - 2H]$^+$**	191.92 ± 0.12	N					
	**[(M)(*L*-His)$_2$(Cu^{II})$_2$ - 3H]$^+$**	Not observed in DTIMS screening; not searched in TWSLIM spectrum.						
**Penicillamine**	**[(M)(*L*-His)(Cu^{II}) - H]$^+$**	173.53 ± 0.06	N					
	**[(M)(*L*-His)(Cu^{II}) + Na - 2H]$^+$**	Not observed in DTIMS screening; not searched in TWSLIM spectrum.						
	**[(M)(*L*-His)$_2$(Cu^{II})$_2$ - 3H]$^+$**	203.52 ± 0.38	N					
**Flurbiprofen**	**[(M)(*L*-His)(Cu^{II}) - H]$^+$**	211.28 ± 0.18	N					
	**[(M)(*L*-His)(Cu^{II}) + Na - 2H]$^+$**	201.18 ± 0.19	N					
	**[(M)(*L*-His)$_2$(Cu^{II})$_2$ - 3H]$^+$**	232.04 ± 0.19	N					
**Metoprolol**	**[(M)(*L*-His)(Cu^{II}) - H]$^+$**	196.81 ± 0.29	Y	194.58	196.88	2.30	1.18%	1.52
	**[(M)(*L*-His)(Cu^{II}) + Na - 2H]$^+$**	200.38 ± 0.19	Y	198.91	200.97	2.10	1.03%	0.97
	**[(M)(*L*-His)$_2$(Cu^{II})$_2$ - 3H]$^+$**	235.84 ± 0.88	N					
**Ofloxacin**	**[(M)(*L*-His)(Cu^{II}) - H]$^+$**	236.58 ± 0.12	N					
	**[(M)(*L*-His)(Cu^{II}) + Na - 2H]$^+$**	237.18 ± 0.16	N					
	**[(M)(*L*-His)$_2$(Cu^{II})$_2$ - 3H]$^+$**	263.60 ± 0.37	N					
**Baclofen**	**[(M)(*L*-His)(Cu^{II}) - H]$^+$**	189.50 ± 0.07	Y	186.77	188.15	1.38	0.74%	0.89
	**[(M)(*L*-His)(Cu^{II}) + Na - 2H]$^+$**	192.77 ± 0.13	N					
	**[(M)(*L*-His)$_2$(Cu^{II})$_2$ - 3H]$^+$**	219.28 ± 0.10	Y	217.43	218.22	0.79	0.36%	0.50
**Chloramphenicol**	**[(M)(*L*-His)(Cu^{II}) - H]$^+$**	211.48 ± 0.19	N					
	**[(M)(*L*-His)(Cu^{II}) + Na - 2H]$^+$**	Not observed in DTIMS screening; not searched in TWSLIM spectrum.						
	**[(M)(*L*-His)$_2$(Cu^{II})$_2$ - 3H]$^+$**	Not observed in DTIMS screening; not searched in TWSLIM spectrum.						
**Ethosuximide**	**[(M)(*L*-His)(Cu^{II}) - H]$^+$**	175.40 ± 0.56	N					
	**[(M)(*L*-His)(Cu^{II}) + Na - 2H]$^+$**	Not observed in DTIMS screening; not searched in TWSLIM spectrum.						
	**[(M)(*L*-His)$_2$(Cu^{II})$_2$ - 3H]$^+$**	205.97 ± 0.17	N					
**Omeprazole**	**[(M)(*L*-His)(Cu^{II}) - H]$^+$**	220.43 ± 1.79	N					
	**[(M)(*L*-His)(Cu^{II}) + Na - 2H]$^+$**	221.53 ± 1.64	N					
	**[(M)(*L*-His)$_2$(Cu^{II})$_2$ - 3H]$^+$**	Not observed in DTIMS screening; not searched in TWSLIM spectrum.						
**Ibuprofen**	**[(M)(*L*-His)(Cu^{II}) - H]$^+$**	203.64 ± 0.07	N					
	**[(M)(*L*-His)(Cu^{II}) + Na - 2H]$^+$**	194.71 ± 0.07	N					
	**[(M)(*L*-His)$_2$(Cu^{II})$_2$ - 3H]$^+$**	225.00 ± 0.07	N					

Figure S7. To validate in-house calculations of resolving power (R_P) and two-peak resolution (R_{P-P}), the peak fitting software PeakLab was used to (**A**) fit Gaussians to experimental data. **Figure S7A** shows this theoretical peak fitting for [(*rac*-Thalidomide)(*L*-His)(Cu^{II}) – $H]^+$. This was repeated for each ternary *L*-His:Cu complex shown in Figure 3, including baclofen, metoprolol, and panthenol. Using data points from the fitted peaks, R_P and R_{P-P} were calculated. Shown in (**B**) is a comparison of in-house R_P and R_{P-P} values to values obtained using PeakLab. PeakLab's results indicate R_{P-P} values slightly lower than in-house and R_P values slightly higher than in-house—though we note that the values are close enough in value that we consider PeakLab results satisfactory validation for our data.

(A)

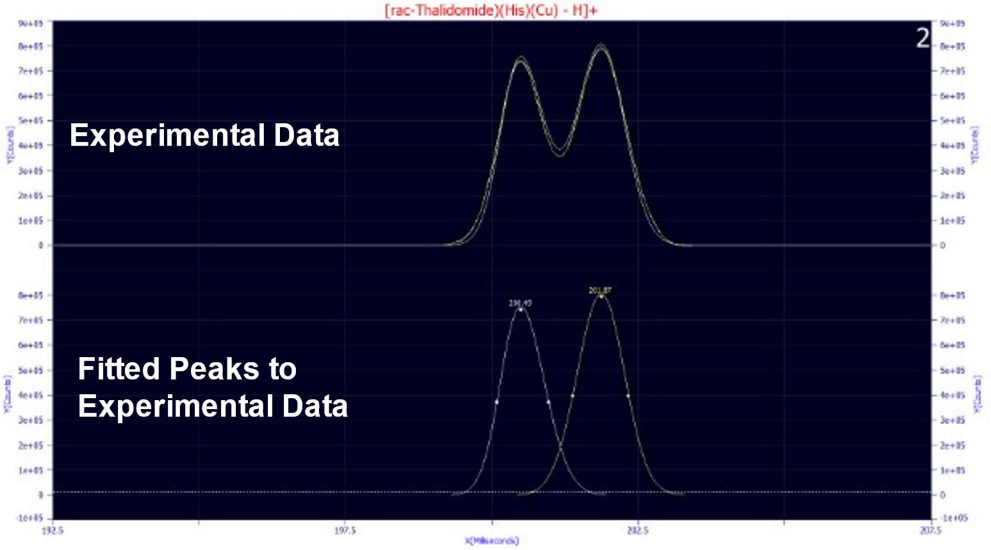

(B) Comparison of Resolving Powers and Two-Peak Resolutions: In-house vs. PeakLab

[(M)(*L*-His)(Cu^{II}) – $H]^+$	R_p		R_{P-P}	
M =	In-house	Peak Lab	In-house	Peak Lab
rac-Baclofen	213, 199	222, 221	0.9	0.8
rac-Metoprolol	223, 220	232, 228	1.5	1.4
rac-Panthenol	228, 201	234, 231	0.7	0.6
rac-Thalidomide	223, 203	228, 212	0.8	0.8

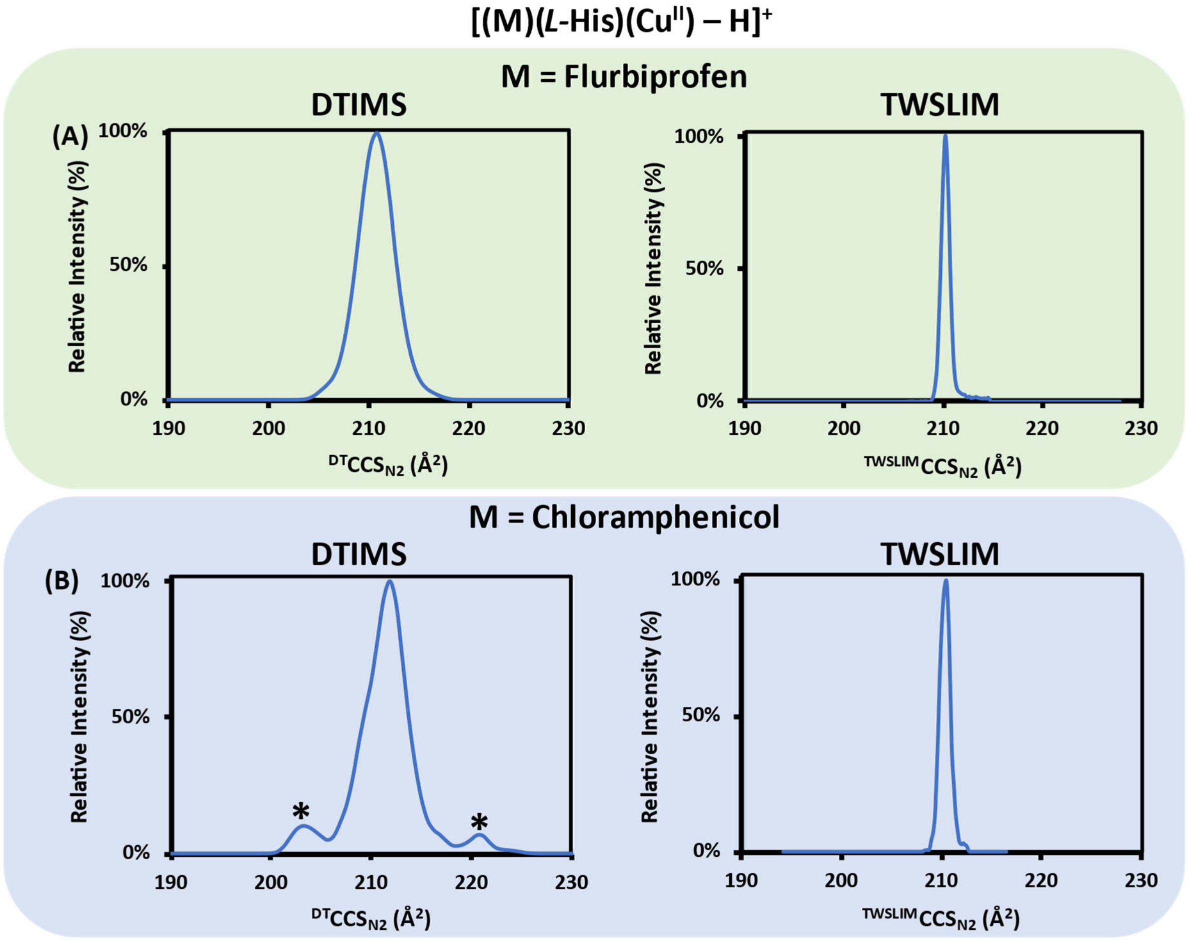

Figure S8. DTIMS and TWSLIM spectra for two selected compounds whose mononuclear complexes (spectra shown) did not exhibit differentiation in HRIM analysis. Compounds include **(A)** flurbiprofen (green panel) and **(B)** chloramphenicol (blue panel). Extraneous mobility features arising from signals of different *m/z* than the targeted analytes are marked with an asterisk (*).

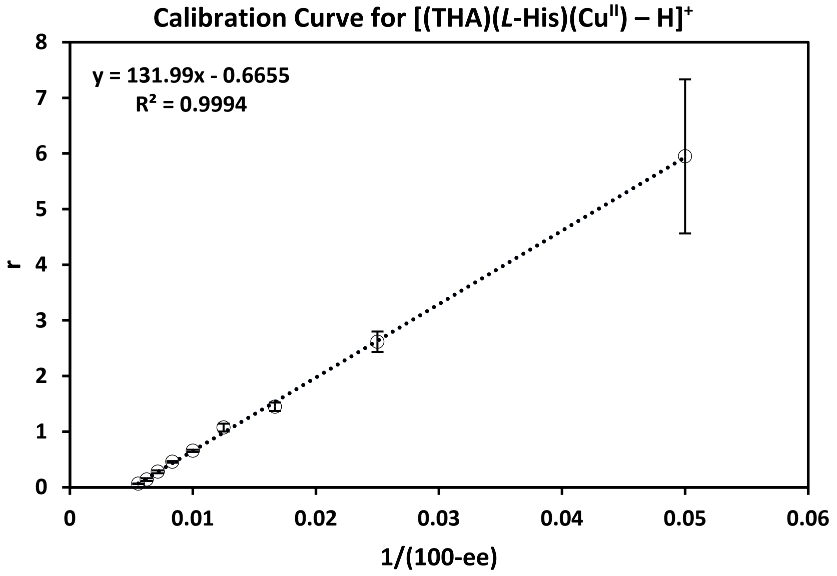

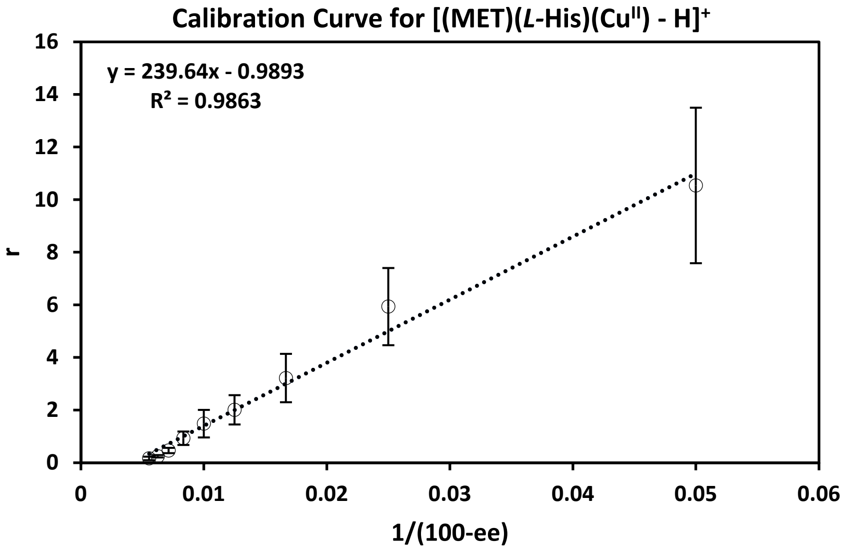

Figure S9. Linear calibration curve obtained relating peak area ratio, *r*, to enantiomeric excess, *ee*. Error bars represent standard error across replicates.

Table S9. Interday replicate measurements for each sample analyzed in thalidomide enantiomeric quantitation experiments. Additionally, peak areas for each enantiomer of thalidomide as well as *r* calculations are included.

Ratio [*S* to *R*]	[*S*] (μM)	[*R*] (μM)	*ee* (*S*)	1/(100-*ee*)	Theoretical (*S*) to (*R*)	Peak area of *S*-THA	Peak area of *R*-THA	(*S*)/(*R*)
1 to 9	10	90	-80	0.00555	0.11	13267	196631	0.07
1 to 9	10	90	-80	0.00555	0.11	12856	229983	0.06
1 to 9	10	90	-80	0.00555	0.11	584266	8386811	0.07
2 to 8	20	80	-60	0.00625	0.25	37199.5	230252	0.16
2 to 8	20	80	-60	0.00625	0.25	21053	240734	0.09
2 to 8	20	80	-60	0.00625	0.25	1192023	7161851	0.17
3 to 7	30	70	-40	0.00714	0.43	52320.5	159712	0.33
3 to 7	30	70	-40	0.00714	0.43	42893	179612	0.24
3 to 7	30	70	-40	0.00714	0.43	1932199	7127259	0.27
4 to 6	40	60	-20	0.00833	0.67	98844	203707	0.49
4 to 6	40	60	-20	0.00833	0.67	74666	167031	0.45
4 to 6	40	60	-20	0.00833	0.67	3348526	7651721	0.44
5 to 5	50	50	0	0.01	1.00	115722	172954	0.67
5 to 5	50	50	0	0.01	1.00	103661	152349	0.68
5 to 5	50	50	0	0.01	1.00	3717832	6023255	0.62
6 to 4	60	40	20	0.0125	1.50	144399	122106	1.18
6 to 4	60	40	20	0.0125	1.50	112550	102508	1.10
6 to 4	60	40	20	0.0125	1.50	3910817	4150539	0.94
7 to 3	70	30	40	0.01667	2.33	142343	91864	1.55
7 to 3	70	30	40	0.01667	2.33	103591	69294	1.50
7 to 3	70	30	40	0.01667	2.33	4922039	3806476	1.30
8 to 2	80	20	60	0.025	4.00	200257	74681	2.68
8 to 2	80	20	60	0.025	4.00	155677	53701	2.90
8 to 2	80	20	60	0.025	4.00	6423605	2858312	2.27
9 to 1	90	10	80	0.05	9.00	165556	29651	5.58
9 to 1	90	10	80	0.05	9.00	142055	16691	8.51
9 to 1	90	10	80	0.05	9.00	6980571	1960092	3.75

Table S10. Average *r* values (*r* = PAR) and average standard errors for use in thaldiomide and metoprolol calibration curves. Average peak area ratio is the average of all interday and intraday *r* values.

**Thalidomide Data Analysis**								
**Ratio [(*S*) to (*R*)]**	***ee* (*S*)**	**1/(100-*ee*)**	**Theoretical (*S*) to (*R*)**	**Average Peak Area (*S*)**	**Average Peak Area (*R*)**	**Average Peak Area Ratio [(*S*)/(*R*)]**	**SD PAR**	**SE PAR**
1 to 9	-80	0.00556	0.11	203463	2937809	0.0643	0.00738	0.00426
2 to 8	-60	0.00625	0.25	416759	2544279	0.138	0.0442	0.0255
3 to 7	-40	0.00714	0.43	675804	2488861	0.279	0.0449	0.0259
4 to 6	-20	0.00833	0.67	1174012	2674153	0.456	0.0251	0.0145
5 to 5	0	0.01	1.00	1312406	2116186	0.655	0.0336	0.0194
6 to 4	20	0.0125	1.50	1389256	1458385	1.07	0.122	0.0704
7 to 3	40	0.01667	2.33	1722658	1322545	1.45	0.132	0.0761
8 to 2	60	0.025	4.00	2259847	995565	2.62	0.320	0.184
9 to 1	80	0.05	9.00	2429394	668812	5.95	2.40	1.39

**Metoprolol Data Analysis**								
**Ratio [(R) to (S)]**	***ee* (R)**	**1/(100-ee)**	**Theoretical (R) to (S)**	**Average Peak Area (R)**	**Average Peak Area (S)**	**Average Peak Area Ratio [(R)/(S)]**	**SD PAR**	**SE PAR**
1 to 9	-80	0.00556	0.11	1769	15808	0.165	0.115	0.0664
2 to 8	-60	0.00625	0.25	4631	19048	0.246	0.0801	0.0465
3 to 7	-40	0.00714	0.43	6632	15402	0.463	0.174	0.100
4 to 6	-20	0.00833	0.67	13184	14048	0.930	0.438	0.253
5 to 5	0	0.01	1.00	20890	19396	1.49	0.904	0.522
6 to 4	20	0.0125	1.50	16659	11349	2.01	0.965	0.557
7 to 3	40	0.01667	2.33	16688	7115	3.22	1.6	0.921
8 to 2	60	0.025	4.00	22262	4790	5.94	2.54	1.45
9 to 1	80	0.05	9.00	27996	3917	10.5	5.12	2.96

Table S11. Comparison of the thalidomide actual *ee* (enantiomeric excess of sample) versus measured *ee* (enantiomeric excess predicted by calibration curve based on average *r* values. The same data is also provided for metoprolol.

Thalidomide Data						
**Sample (*S* to *R*)**	***ee*, *S* (actual)**	**1/(100-*ee*) (actual)**	**(*S*)/(*R*)**	**1/(100-*ee*) (measured)**	***ee* (*S*) (measured)**	**Residual**
1 to 9	-80.00%	0.00556	0.0643	0.00552	-80.85%	0.85%
2 to 8	-60.00%	0.00625	0.138	0.00609	-64.17%	4.17%
3 to 7	-40.00%	0.00714	0.279	0.00716	-39.72%	-0.28%
4 to 6	-20.00%	0.00833	0.456	0.00850	-17.62%	-2.38%
5 to 5	0.00%	0.01	0.6555	0.0101	0.09%	-0.09%
6 to 4	20.00%	0.0125	1.0742	0.0132	24.13%	-4.13%
7 to 3	40.00%	0.01667	1.447	0.0161	37.54%	2.46%
8 to 2	60.00%	0.025	2.616	0.0249	59.78%	0.22%
9 to 1	80.00%	0.05	5.948	0.0501	80.04%	-0.04%
Racemic	0.00%	0.01	0.583	0.00945	-5.73%	5.73%

Metoprolol Data						
**Sample (*R* to *S*)**	***ee*, *R* (actual)**	**1/(100-*ee*) (actual)**	**(*R*)/(*S*)**	**1/(100-*ee*) (measured)**	***ee* (*R*) (measured)**	**Residual**
1 to 9	80.00%	0.00556	0.165	0.00482	-107.67%	27.67%
2 to 8	60.00%	0.00625	0.246	0.00516	-93.96%	33.96%
3 to 7	40.00%	0.00714	0.463	0.00606	-65.00%	25.00%
4 to 6	20.00%	0.00833	0.930	0.008	-24.83%	4.83%
5 to 5	0.00%	0.01	1.485	0.0103	3.13%	-3.13%
6 to 4	20.00%	0.0125	2.008	0.0125	20.07%	-0.07%
7 to 3	40.00%	0.01667	3.219	0.0176	43.06%	-3.06%
8 to 2	60.00%	0.025	5.935	0.0289	65.39%	-5.39%
9 to 1	80.00%	0.05	10.540	0.0481	79.22%	0.78%
Racemic	0.00%	0.01	1.472	0.0103	-2.46%	2.46%

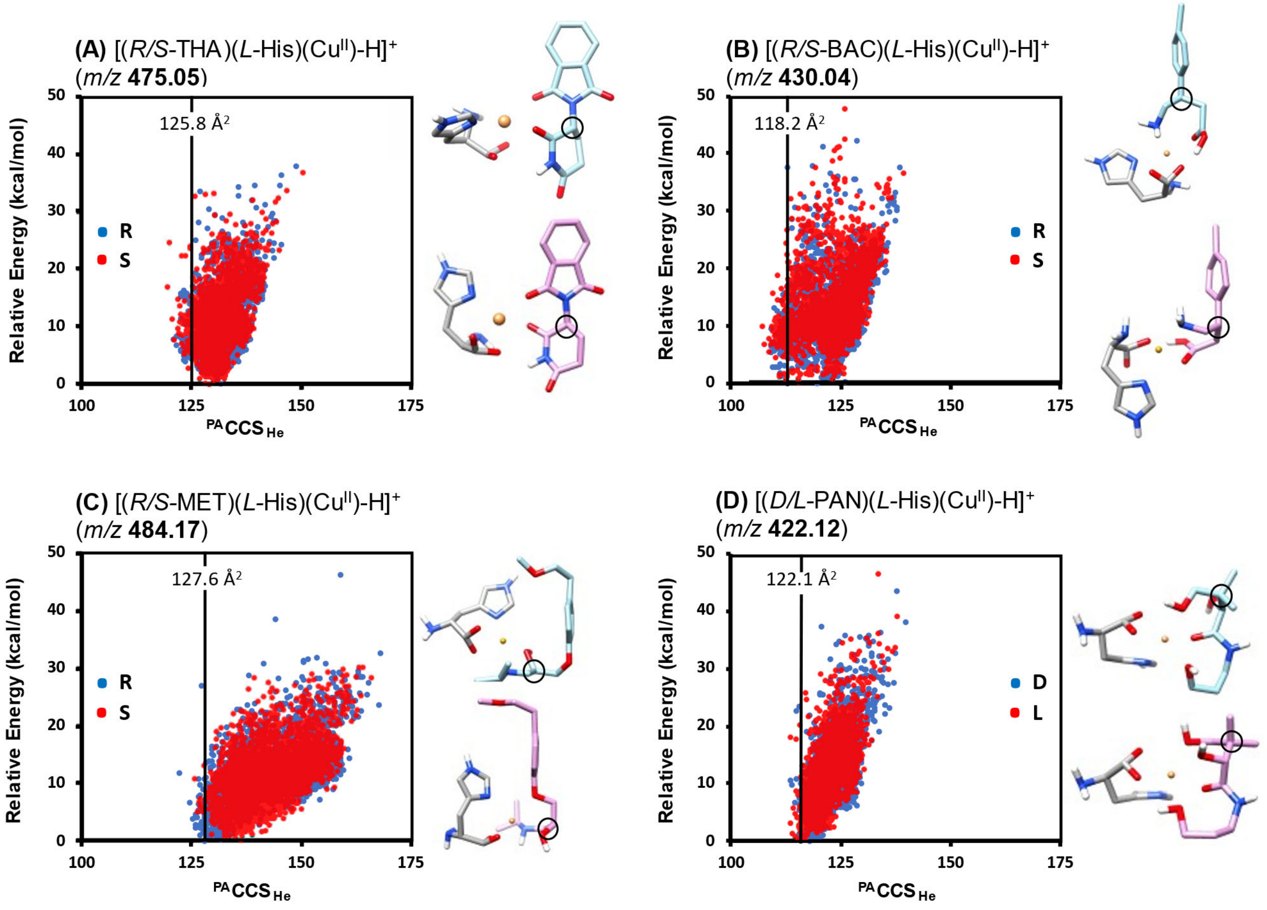

Figure S10. Theoretical helium conformational space plots. Theoretical calculations were performed for mononuclear $[(M)(L\text{-His})(Cu^{II}) - H]^+$ complexes which were differentiated in HRIM analysis, namely **(A)** *R/S*-thalidomide, **(B)** *R/S*-baclofen, **(C)** *R/S*-metoprolol, and **(D)** *D/L*-panthenol. Structures shown represent the average structure of the 600 lowest energy structures for each M:*L*-His:Cu^{II} complex, where circled in black are chiral centers for each average structure. While the theoretical CCS calculations were performed in helium, structural and separation trends gleaned from these helium observations are applicable to experimental TWSLIM observations. Aligned to theoretical conformational space plots are experimental $^{DT}CCS_{He}$ values (black vertical lines). To simplify the plots, the $^{DT}CCS_{He}$ provided for each plot is the average of helium measurements for all enantiomers for a given drug. To illustrate, 125.8 Å^2 was calculated for thalidomide by averaging the $^{DT}CCS_{He}$ values for *R*, *S*, and *rac*-thalidomide mononuclear complexes.

Table S12. Drift tube CCS measurements obtained in helium drift gas ($^{DT}CCS_{He}$) for successfully-separated mononuclear copper complexes of the stoichiometry [(M)(*L*-His)(Cu^{II}) – H]$^+$, where M is an analyte ligand of a given chirality. Because helium contributes less to measured CCS values, helium-based CCS measurements are in better agreement with computational results and are thus used for alignment of theoretical data.

**Helium CCS Values for [(M)(*L*-His)(Cu^{II}) - H]$^+$**		
**M=**	***m/z* [Da]**	**$^{DT}CCS_{He}$ [Å^2]**
R-Thalidomide	475.05	125.4
rac-Thalidomide	475.05	126.4
S-Thalidomide	475.05	125.7
R-Metoprolol	484.17	127.8
rac-Metoprolol	484.17	129.2
S-Metoprolol	484.17	125.8
R-Baclofen	430.04	117.6
rac-Baclofen	430.04	118.7
D-Panthenol	422.12	122.4
rac-Panthenol	422.12	121.8
